# Formation and subdivision of the head field in the centipede *Strigamia maritima*, as revealed by the expression of head gap gene orthologues and *hedgehog* dynamics

**DOI:** 10.1186/s13227-017-0082-x

**Published:** 2017-10-23

**Authors:** Vera S. Hunnekuhl, Michael Akam

**Affiliations:** 1 Laboratory for Development and Evolution, Department of Zoology, University of Cambridge, Downing Street, Cambridge, CB23EJ UK; 2 Department of Evolutionary Developmental Genetics, Georg-August-Universität Göttingen, Caspari Haus, Justus-von-Liebig-Weg 11, 37077 Göttingen, Germany

## Abstract

**Background:**

There have been few studies of head patterning in non-insect arthropods, and even in the insects, much is not yet understood. In the fly *Drosophila* three head gap genes, *orthodenticle* (*otd*), *buttonhead* (*btd*) and *empty spiracles* (*ems*) are essential for patterning the head. However, they do not act through the same pair-rule genes that pattern the trunk from the mandibular segment backwards. Instead they act through the downstream factors *collier* (*col*) and *cap*‘*n*’*collar* (*cnc*), and presumably other unknown factors. In the beetle *Tribolium*, these same gap and downstream genes are also expressed during early head development, but in more restricted domains, and some of them have been shown to be of minor functional importance. In the spider *Parasteatoda tepidariorum*, *hedgehog* (*hh*) and *otd* have been shown to play an important role in head segmentation.

**Results:**

We have investigated the expression dynamics of *otx* (*otd*), *SP5*/*btd*, *ems*, and the downstream factors *col*, *cnc* and *hh* during early head development of the centipede *Strigamia maritima*. Our results reveal the process of head condensation and show that the anteroposterior sequence of specific gene expression is conserved with that in insects. *SP5*/*btd* and *otx* genes are expressed prior to and during head field formation, whereas *ems* is not expressed until after the initial formation of the head field, in an emerging gap between *SP5*/*btd* and *otx* expression. Furthermore, we observe an early domain of *Strigamia hh* expression in the head field that splits to produce segmental stripes in the ocular, antennal and intercalary segments.

**Conclusions:**

The dynamics of early gene expression in the centipede show considerable similarity with that in the beetle, both showing more localised expression of head gap genes than occurs in the fly. This suggests that the broad overlapping domains of head gap genes observed in *Drosophila* are derived in this lineage. We also suggest that the splitting of the early *hh* segmental stripes may reflect an ancestral and conserved process in arthropod head patterning. A remarkably similar stripe splitting process has been described in a spider, and in the *Drosophila* head *hh* expression starts from a broad domain that transforms into three stripes.

**Electronic supplementary material:**

The online version of this article (doi:10.1186/s13227-017-0082-x) contains supplementary material, which is available to authorized users.

## Background

The mechanisms that specify the head segments of arthropods are fundamentally different from those that operate in the trunk [1–4]. Some of the genes that define segment boundaries in the trunk are also used in the head (*engrailed*, *wingless*, *hedgehog*), but how the expression of these segment polarity genes is linked to upstream axial patterning remains unclear.

Currently, most insights into arthropod head patterning are based on studies from the fly *Drosophila melanogaster*. A gradient of the maternally provided anterior morphogen bicoid (bcd) in the blastoderm stage embryo [5] leads to the activation of the so-called head gap genes *orthodenticle* (*otd*), *empty spiracles* (*ems*) and *buttonhead* (*btd*) in broad overlapping domains. They were termed gap genes because mutations in these genes delete a coherent block of specific head segments [6–10]. *Sloppy paired* has also been proposed as a head gap gene [11], but its role in head development remains unclear. It is not known whether these genes are direct targets of bcd, but *cis*-acting elements that contain bcd binding sites have been identified upstream of *ems* and *btd* [8, 9].

In *Drosophila*, the domains of expression of these head gap genes are out of phase by one segment [1, 2, 6]. Each factor is required for the correct establishment of a subset of segmental stripes of *wingless* (*wg*) and/or *hedgehog* (*hh*) expression in the head [12]. It has not yet been fully elucidated whether there is direct control of segment polarity gene expression by the head gap genes or whether their effects are transduced through second level mediators that would replace the pair rule patterning system that acts in the *Drosophila* trunk but not in the head (see [1]).

The whole head segmentation mechanism in *Drosophila* is likely to be in at least some respects specific to the higher *Diptera*: bcd as a major anterior determinant is only present in the higher flies [13–15]. Different maternal signals are used in other insects [16, 17]. In many arthropods, the embryo is patterned after cellularisation, and any link between maternal coordinates and embryonic patterning remains unknown. Since the earliest patterning mechanism is so specialised in *Drosophila*, it is also questionable whether the downstream mechanism of gap gene patterning is present in the same way in other arthropods.

More recently, the beetle *Tribolium castaneum* has been established as a second model for insect head patterning. Work in *Tribolium* has shown that orthologues of the *Drosophila* gap genes *otd*, *ems* and *btd* are expressed during early head development [18–20], but their expression domains are not as broadly overlapping as they are in *Drosophila*. RNAi mediated knockdown of *ems* and *btd* had no (for *btd*) or only rather subtle (for *ems*) effects when compared to the gap gene mutant phenotypes in *Drosophila*. Only the knockdown of *otd* produced a severe head phenotype [20]. RNAi knockdown of *ems* and *otd* orthologues in the milkweed bug *Oncopeltus fasciatus also* gave mild phenotypes. No *btd* orthologue could be isolated from this species [21]. In both *Tribolium* and *Oncopeltus*, these mild phenotypes may be due to limitations of the RNAi method and need to be confirmed by gene knockout. Regardless of this, the results suggest that orthologues of *Drosophila* head gap genes are expressed during early head development in other insects, but their role may be rather different.

Another transcription factor of interest for insect head patterning is *collier* (*col*), which acts downstream of the head gap genes *btd* and *ems* in the fly. *Col* was the first intermediately acting factor to be identified in *Drosophila* head segmentation [22]. It directly controls the expression of segment polarity genes in the intercalary segment [22, 23]. It is also required for correct expression of *cap*‘*n*’*collar*, a factor that specifies mandibular identity [22, 24]. Furthermore, *col* is repressed at its posterior limit by the trunk pair rule gene *even*-*skipped*, hence taking input from both the head and the trunk patterning system [22]. Expression of *col* in the pre-mandibular region is conserved in other insects and in the millipede *Glomeris marginata* and has been discussed as being responsible for the appendage-less phenotype of the intercalary segment in insects and myriapods [25, 26]. However, the lack of a homeotic phenotype after removal of *col*, and its function in head segmentation in *Drosophila* [22, 26, 27], rather speak in favour of a crucial role in head segmentation and integration of head and trunk patterning that might be conserved in myriapods. Therefore, we included *col* in our analysis of head patterning genes in the centipede.

Further insights into arthropod head patterning come from work on the spider *Parasteatoda tepidariorum* (formerly called *Achaearanea tepidariorum*). Here, the signalling factor *hedgehog* (*hh*) is dynamically expressed in a cephalic domain that splits into three segmental stripes [3, 4]. Subdivision of a cephalic *hh* domain into ocular, antennal and intercalary segmental stripes also happens in *Drosophila* head segmentation [28], though this process has been little studied. A study in the millipede *G. marginata* has shown that the anterior-most *hh* stripes, belonging to the ocular and the antennal segment, are also generated by a stripe splitting event [29]. These similar patterns suggest that a domain-splitting mechanism, rather than a sequential addition of segments [4], might generally characterise arthropod head segmentation. We ask whether subdivision of an early cephalic *hh* domain also occurs in this centipede, and how this domain and resulting subdomains relate to the expression of other early expressed head patterning genes.

We chose to study the centipede *Strigamia maritima* to provide information on head patterning from another distant outgroup to the insects and to facilitate an evolutionary comparison of head patterning across the arthropods. Our work complements the recent work on head patterning in another myriapod, the millipede *G. marginata*, where *otd* and a putative *btd* orthologue were found to be anteriorly expressed [30], and the work on head patterning in the spider *P. tepidariorum* discussed above. Only little data are available on gene expression during head patterning in crustaceans [31, 32].

The *S. maritima* genome has been sequenced and extensively annotated [33]. There is a comprehensive modern description of its embryology [34], and some developmental processes, including neurogenesis and trunk segmentation, have already been studied in considerable detail [35–38]. Research on *Strigamia* has so far been limited to descriptive work (as it has in all myriapods); nevertheless, the detailed analysis of gene expression, and especially the spatial and temporal relationship of expression in relation to the process of morphogenesis, has provided significant insight into patterning mechanisms and possible hierarchical interactions of the factors involved [37, 38].

We identified in the *Strigamia* genome candidates for orthologues of *buttonhead* (*SP5*), *empty spiracles*, *orthodenticle*, *collier* and *cap*‘*n*’*collar*, and characterised their genomic organisation. We visualised the spatial and temporal dynamics of the expression of these genes and the degree of overlap of their domains and analysed how their expression relates to the morphodynamics of head field formation and to the first expression of the segment polarity gene *hedgehog*. While we find that all of the investigated factors are anteriorly expressed during early centipede development, we find that the combinatorial pattern of the investigated genes in the centipede is more similar to that seen in the beetle *Tribolium* than it is to that of *Drosophila*. Hence, we conclude that some aspects of the head gap gene patterning system have evolved in the fly lineage. We additionally show two rounds of subdivision of an early cephalic *hh* domain, which is reminiscent of the *hh* dynamics described in the spider [4], although the precise temporal dynamic is different. We find that the resulting ocular, antennal and intercalary segmental stripes of *hh* expression co-localise with ocular *otx* and antennal/intercalary *SP5*/*btd* expression, respectively.

## Results

### The centipede orthologues of insect head patterning genes

The sequenced genome of *S. maritima* contains orthologues of most known insect head patterning genes. We found one likely *buttonhead*/*SP5* orthologue (among three SP family genes), three *orthodenticle*/*otx* genes (*otx*-*A*, *otx*-*B and otx*-*C*), one *empty spiracles* (*ems*), one *collier* (*col*) and two *cap*‘*n*’*collar* (*cnc1* and *cnc2*) genes (see also Additional file 1). In addition, we identified two orthologues of the segment polarity factor *hedgehog* (*hh1 and hh2*). Gene models of all these factors can be found at http://metazoa.ensembl.org/Strigamia_maritima/. A list of genes including accession numbers, used primer sequences and probe lengths is provided in Additional file 1: Table S1.

### SP5/*buttonhead* as a marker for early head patterning

The *buttonhead* (*btd*) gene is a zinc finger domain transcription factor that was first characterised as a head patterning gene in *Drosophila* [6]. It is the orthologue of the vertebrate SP5 genes within the SP family of transcription factors (see [39]). The likely orthologous *Strigamia* gene is here referred to as *SP5*/*btd* (see Additional file 1 and Additional file 2: Fig. S1 and Additional file 3: Fig. S2 for the proposed classification of the three centipede SP genes). This gene is a valuable marker for early patterning of the head (see below). There are two other SP family genes in *Strigamia*, all localised in a single chromosomal cluster with *SP5*/*btd*. Orthologies within the SP family are not completely clear, but these other genes likely represent an *SP6*-*9* orthologue and an *SP1*-*4* orthologue (see Additional file 2: Fig. S1).

**Fig. 1:**
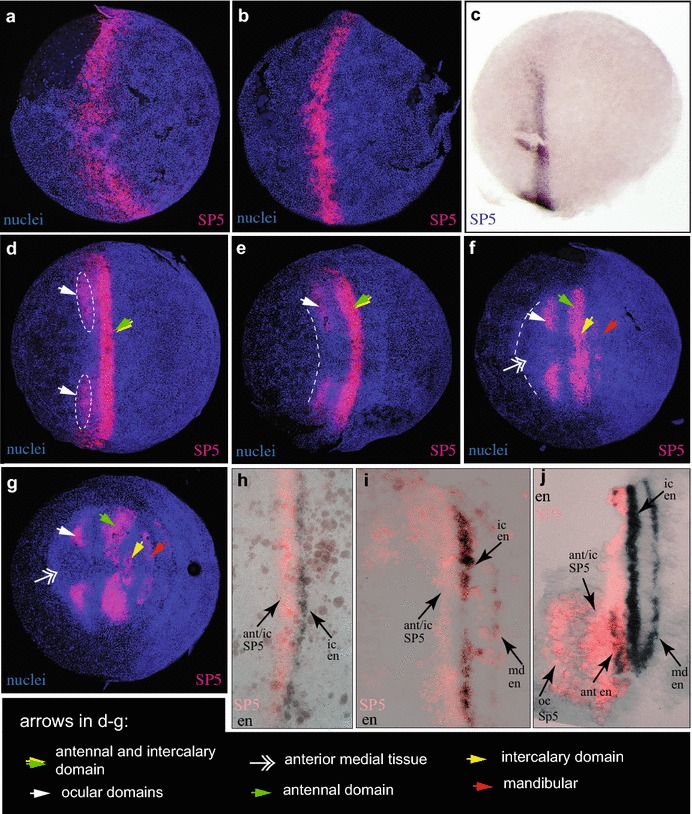
Expression of *SP5*/*btd* in early Strigamia development. All panels show ventral views, except (**c**), which is a lateral view. **a** (stage 2.1) A broad ring of *SP5*/*btd* expression surrounds the uniform blastoderm. **b** (stage 2.2, early) *SP5*/*btd* expression lies at the transition between the single layered blastoderm in the anterior and denser (multi-layered) nuclei in the posterior. **c** (stage 2.2, early) Lateral view of similar staged embryo as in (**b**): the ring of *SP5*/*btd* expression has opened dorsally. **d** (stage 2.2, late) The initial ring of *SP5*/*btd* expression has condensed to a domain situated at the posterior of the emerging head field; this domain (green arrowhead) marks the future antennal and intercalary tissue. Bilateral (ocular) expression domains (circled, white arrowheads) emerge anteriorly adjacent to this domain. **e** (stage 2.3, early) Ocular domains detach from the more posterior domain. A dashed line marks the anterior margin of the condensed head field. **f** (mid-stage 2.3) The head primordium is now clearly defined; the anterior medial region (double headed arrow) occupies the anterior-most part of the head. Mandibular *SP5*/*btd* expression appears posterior to the antennal/intercalary *SP5*/*btd* domain, which is still continuous. **g** (stage 3.1) The antennal and intercalary regions of *SP5*/*btd* expression have separated. **h**–**j** Enlargements of the ventral parts of embryos stained for *SP5*/*btd* and *engrailed* (*en*). **h** (stage 2.2) Early *SP5*/*btd* expression lies directly anterior to the first (intercalary) stripe of *en* expression. **i** (stage 2.2, late) Intercalary *en* expression is directly posterior to antennal/intercalary *SP5*/*btd* expression; the mandibular en stripe emerges more posteriorly. **j** (stage 2.3 early) Antennal *en* expression appears within the main *SP5*/*btd* domain. *oc* ocular, *int* intercalary, *ant* antennal, *md* mandibular

The *Strigamia SP5*/*btd* gene is expressed before the head field becomes apparent in a broad domain at the anterior edge of the forming embryo. Subsequently, as the head field develops, the expression resolves into three domains belonging to the ocular, antennal and intercalary segments (see below). The *SP6*-*9* orthologue is expressed in a similar pattern to *SP5*/*btd* in the anterior head (compare Fig. 1, and Additional file 4: Fig. S3 B, C), but first turns on slightly later. Following early head patterning, both of these genes are also expressed in a segmentally re-iterated pattern throughout the trunk (see Fig. 1g and Additional file 4: Fig. S3 D). The third SP family gene, *SP1*-*4* is widely expressed in a near-ubiquitous pattern throughout later embryonic development (Additional file 4: Fig. S3 E–H), a pattern similar to that observed for *SP1*-*4* orthologues in other arthropods [39]. We have not studied the expression of these other family members in detail.

#### *SP5*/*btd* expression and initial formation of the head field at the ventral side

**Fig. 2:**
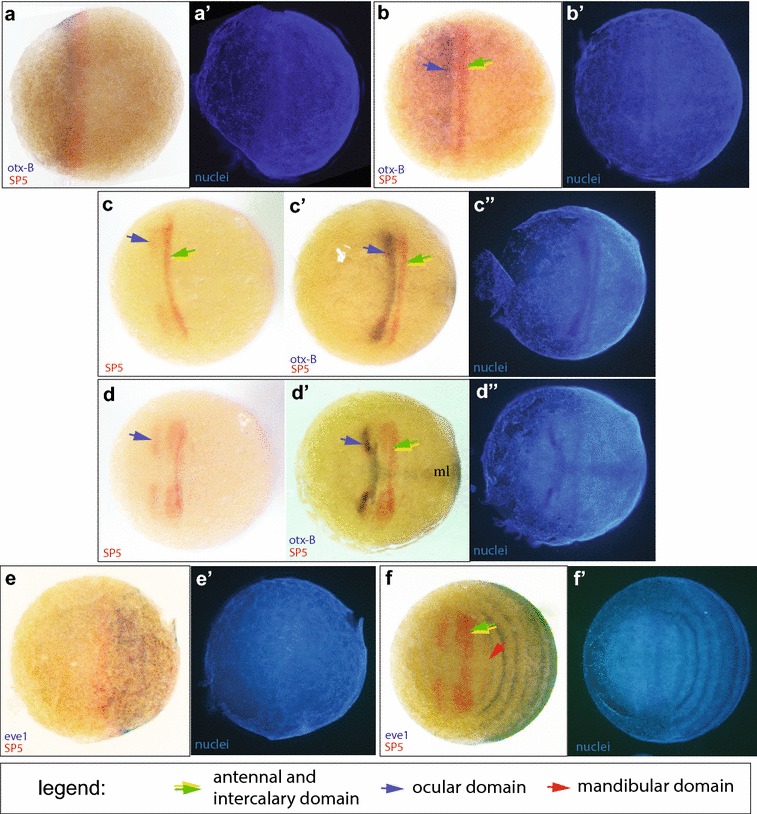
*SP5*/*btd* expression in conjunction with *otx*-*B* (**a**–**d**) and *eve1* (**e**, **f**). All panels show ventral views. **a** (stage 2.2, early) An anterior cap of *otx*-*B* expression is directly anteriorly adjacent to the early ring-domain of *SP5*/*btd*. **b** (stage 2.3, early) A narrow gap has emerged between the antennal/intercalary *SP5*/*btd* expression and the more anterior *otx*-*B* expression. **c** (early stage 2.3) Photograph of only the *SP5*/*btd* stain shows ocular patches of *SP5*/*btd* expression anteriorly to antennal/intercalary domain. **c**′ Same specimen as **c** after *otx*-*B* stain, the strong part of the *otx* expression overlaps with ocular *SP5*/*btd* expression. *Otx*-*B* expression more anteriorly in the condensing head field (compare Additional file 4: figure S3) is barely visible due to reduced sensitivity of double stainings. **d** (stage mid-2.3, *SP5*/*btd* only) **d**′ (same specimen as **d**) Ocular *otx*-*B* expression is subdivided into lateral patches and a medial domain, the distance between ocular *otx*-*B* and antennal/intercalary *SP5*/*btd* expression has increased. **e** (stage 2.2) Broad expression of *eve1* lies posteriorly adjacent to *SP5*/*btd* expression. **f** (stage 2.3) The posterior dynamic expression of *eve1* resolves into single stripes of which the first lies directly posterior to the mandibular segmental *SP5*/*btd* expression (red arrowhead)

The first visible sign of axial patterning in *Strigamia* is the transition from a uniform blastoderm to a blastoderm in which the posterior half is characterised by a higher density of nuclei. Expression of *SP5*/*btd* starts at this early blastoderm stage (2.1–2.2; all stage references according to [34]), while the cell distribution is still largely uniform. *SP5*/*btd* expression appears as a broad ring that spans the whole circumference of the egg (Fig. 1a). The ring is located directly anterior to the early expression of *even*-*skipped*-*1* (*eve1*) (Fig. 2e), which is expressed throughout the posterior half of the blastoderm. *eve1* expression subsequently resolves into stripes, the most anterior of which is assigned to the posterior mandibular segment (Fig. 2f; and see [37]). Hence, the *SP5*/*btd* expressing part of the blastoderm is likely to give rise to segments directly anterior of the mandibular, which are the intercalary and the antennal segment. Note that the mandibular domain of *SP5*/*btd* appears de novo (see Fig. 1f), posterior to this initial domain.

The head rudiment forms as cells in the anterior half of the blastoderm condense ventrally and posteriorly to form a visible thickening [34]. As this condensation begins, the early ring of *SP5*/*btd* expression narrows in the A/P axis (Fig. 1b), and opens dorsally (Fig. 1c), becoming restricted to the ventral half of the egg. *SP5*/*btd* expression initially marks the transition between the denser nuclei of the forming head field, and the less condensed tissue that lies anteriorly to it (Fig. 1b).

Subsequently, more condensed tissue appears anterior to the primary *SP5*/*btd* domain (Fig. 1d). At the same time a bilateral pair of expression patches emerges directly anterior to, and initially connected with, the main stripe of *SP5*/*btd* expression (encircled areas in Fig. 1d). A little later these patches separate completely from the more posterior domain (Fig. 1e–g). The anterior-lateral position of these detached *SP5*/*btd* expression domains clearly assigns them to the ocular region, while the primary stripe of *SP5*/*btd* marks the antennal and intercalary segment (see below). Subsequently, further condensed material appears anterior to the ocular patches of *SP5*/*btd* expression. This will become the anterior medial region of the head (see Fig. 1f, double headed arrow). For a characterisation of the anterior medial region in the centipede see [40].

At face value, these observations suggest that the antennal and intercalary territory is the first region of the head to condense, followed by the ocular region and then the anterior medial region. However, this interpretation assumes that *SP5*/*btd* expression is a marker for stable populations of cells and does not dynamically shift across cell populations. This remains to be shown.

After the ocular patches have detached, the lateral extent of *SP5*/*btd* expression continues to narrow and the distance between the ocular patches and the more posterior stripe increases (Fig. 1d–f).

#### The major stripe of *SP5*/*btd* expression marks the antennal and intercalary segments

Based on its shape and position, and on comparison with expression of the segmental marker gene *engrailed* (*en*) (Fig. 1h–j), it is evident that the posterior *SP5*/*btd* stripe encompasses the future antennal and anterior intercalary segment. The earliest expression of *en* belongs to the intercalary segment and appears posteriorly adjacent to the early stripe of *SP5*/*btd* expression (compare single stain in Fig. 1b and double *SP5*/*btd*- *en* stain in Fig. 1h). Mandibular *en* expression appears posteriorly of the first *en* stripe well outside the early *SP5*/*btd* domain (Fig. 1i, j), whereas antennal *en* expression appears within the domain (Fig. 1j). In this antennal and intercalary region, *SP5*/*btd* expression appears before the onset of *engrailed* expression.

#### A later phase of *SP5*/*btd* and *SP6-9* expression follows segment formation

From stage 3 onwards, the expression of both *SP5* and *SP6*-*9* appears more posteriorly, in a segmentally re-iterated pattern in the mandibular and more posterior segments (Fig. 1g, Additional file 4: Fig. S3 D). This more posterior expression follows the first appearance of *en* and other segmental markers in these segments, implying that it is not instructive for segment patterning in this region. We assume that this later expression marks the start of a second phase of segmental *SP5*/*btd* expression that might have a tissue specific patterning role in the more posterior segments after their formation.

*SP5*/*btd* in *Strigamia* is continuously expressed in the head rudiment, and its expression makes visible the dynamics of head field formation and subdivision. Therefore, we used *SP5*/*btd* as a reference marker to provide precise staging information, and against which to compare the expression of other head genes.

#### Simultaneous expression of two closely linked centipede *otx* genes

Analysis of the *Strigamia* genome revealed the presence of two closely linked *orthodenticle* (*otd*/*otx*) paralogues (see Additional file 3: Fig. S2 C). Expression of *otx*-*A* was previously characterised within the anterior head of *Strigamia* [41, 42]. We found that *otx*-*B* is expressed in a very similar pattern to *otx*-*A* throughout development, suggesting that the closely linked genes are co-regulated (see Additional file 5: Fig. S4 A, B for single expression of *otx*-*B*). We used a probe against *otx*-*B* in double stains with *SP5*/*btd* and *hh*. A third *otx* gene identified in the *Strigamia* genome, *otx*-*C*, did not show any detectable expression pattern during early development.

#### An emerging gap between ocular *otx* and antennal/intercalary *SP5*/*btd* expression

*otx*-*B* is expressed in the ventral anterior part of the early blastoderm, directly anterior to the ring of *SP5*/*btd* expression with no or minimal overlap (stage 2.2, Fig. 2a). But even at this early stage, we have seen no embryo in which it spans the whole egg circumference, as *SP5*/*btd* expression does. Expression is stronger in the most posterior part that is adjacent to *SP5*/*btd* expression than in the more anterior blastoderm, perhaps reflecting cell density.

Only a little later, by early stage 2.3, a gap emerges between the more posterior domain of *SP5*/*btd* domain and *otx*-*B* expression (Fig. 2b). The strong domain of *otx*-*B* expression is now found in the ocular segment where it overlaps with the ocular expression of *SP5*/*btd* (Fig. 2c, c′, d, d′). *otx*-*B* is also weakly expressed anterior to the ocular domain throughout the condensed head field. This weaker anterior expression is not clearly visible in the double stainings against *SP5*/*btd* and *otx*-*B*, but is seen in single in situ stainings (see Additional file 4: Fig. S4 A, B for *otx*-*B*, and [41, 42] for *otx*-*A*).

As the head condenses further the distance between *otx* expression and antennal/intercalary *SP5*/*btd* expression increases (Fig. 2c′, d′). The ocular *otx* expression domain becomes subdivided into a medial part and two slightly more anterior-lateral patches that overlap with ocular *SP5*/*btd* expression (Fig. 2d, d′).

In summary, the *otx*-*A* and *otx*-*B* genes are broadly expressed at the blastoderm stage before the head field condenses, in the anterior part of the embryo. Expression then becomes restricted to the condensing head field.

#### *empty spiracles* is not an early expressed gap-like gene

**Fig. 3:**
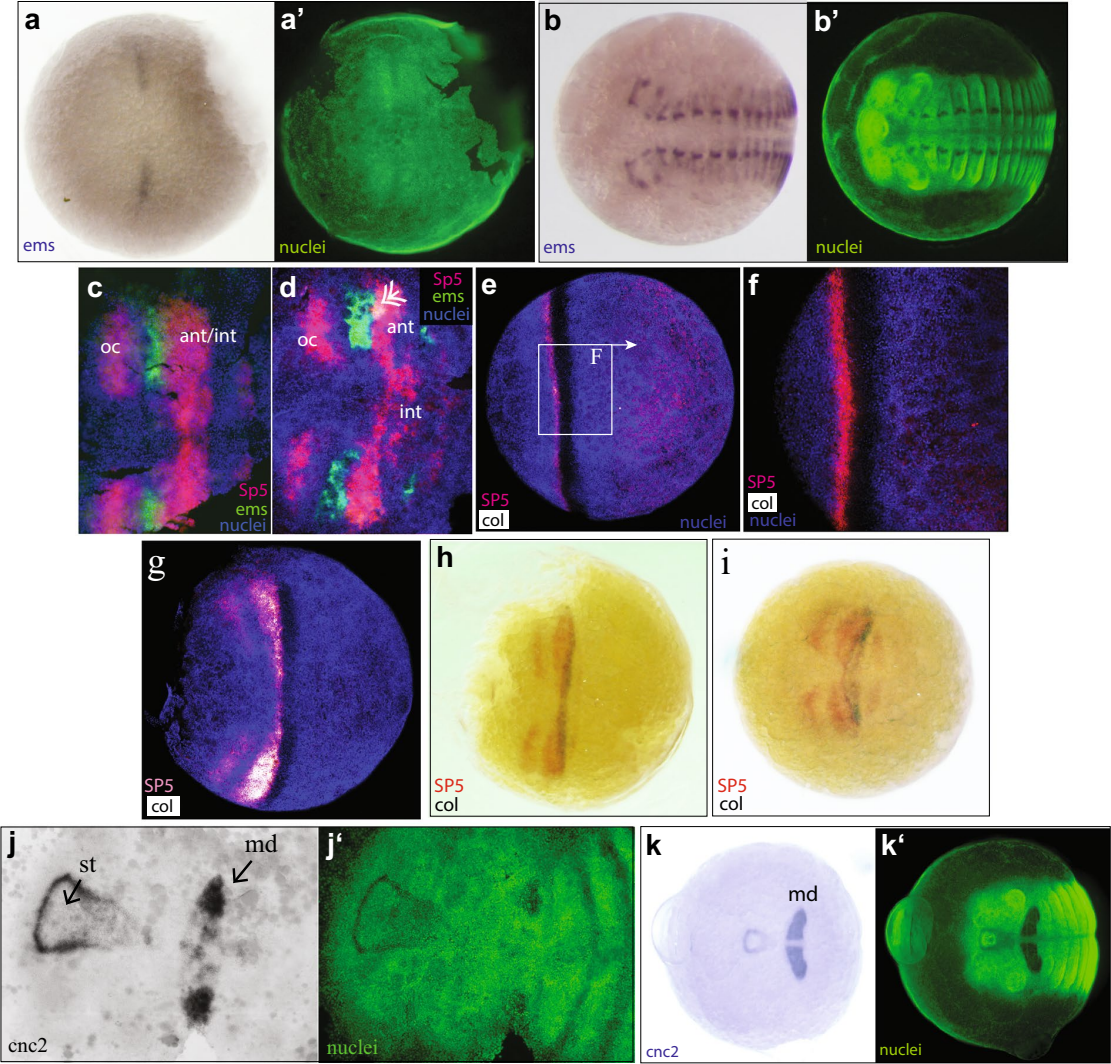
Expression of *ems* (**a**–**c**), *col* (**e**–**i**) and *cnc2* (**j**, **k**) singly (**a**, **b**, **j**, **k**) or in combination with *SP5*/*btd* (**c**–**i**). All panels show ventral views. **a**′, **b**′, **j**′, **k**′—nuclear stain of the same embryos **a** (stage 2.3, early) First expression of *ems* in a pair of bilateral domains. **b** (stage 4.2) Late segmental expression of *ems*. **c** (stage 2.3, early) *ems* expression lies anteriorly adjacent to the antennal part of the *SP5*/*btd* expression, hence located in the anterior antennal segment. **d** (stage 2.3, late) Overlap of *ems* and *SP5*/*btd* in the lateral part of the antennal domain (double headed arrow). **e** (stage 2.2, late), enlarged in **f**. First *col* expression (dark shading of nuclear stain by the BM-purple substrate) overlaps with the early expression of *SP5*/*btd* but is located a few cell widths more posterior. **g** (stage 2.3, early) *col* expression overlaps the posterior of *SP5*/*btd* expression, as *SP5*/btd becomes restricted to the condensing head field. **h** (stage mid-2.3) The *col* domain has narrowed down and is posteriorly included in the antennal/intercalary *SP5*/*btd* domain. **i** (stage 2.3, late) The *col* domain has split into an antennal and an intercalary stripe. **j** (stage 3.2) First appearance of *cnc2* expression in a stomodaeal domain and within the mandibular segment. **k** (stage 4.2) Late segmental and stomodaeal expression of *cnc2*. st = developing stomodaeum, md = mandibular segment

Expression of the *Strigamia empty spiracles* gene (*ems*) was not detected at early blastoderm stage prior to head condensation. First expression was found at early stage 2.3 (Fig. 3a, c) in two small bilateral domains that are located directly anterior to antennal *SP5*/*btd* expression (Fig. 3c, d). These first domains of *ems* expression localise in the emerging gap between ocular *otx* expression and antennal/intercalary *SP5*/*btd* expression. At its posterior edge, *ems* expression overlaps in a few cells with antennal *SP5*/*btd* expression (Fig. 3c, d). With further development, the region of overlap becomes a bit larger but is always restricted to the lateral part of the segment (Fig. 3c, d). *ems* is then expressed in a re-iterated fashion, marking a part of the ectoderm in every segment (Fig. 3b). The anterior antennal part of this segmentally repeated pattern is continuous with the earliest expression of *ems*. Notably *ems* is never expressed in the most anterior head, anterior to the antennal segment.

In summary, *ems* is expressed in the anterior head, but its late appearance and the segmentally repeated pattern does not suggest a specific, instructive function in the early specification of the head.

#### *collier* is expressed early overlapping with *SP5* and then becomes restricted to the anterior intercalary segment

Crozatier et al. [22] characterised *collier* (*col*) as a second level mediator between insect head gap genes and segment polarity genes, with a role in the intercalary and anterior mandibular segment of insects. In *Strigamia* expression of *col* is first detected at the earliest stage of head condensation (Fig. 3e, f), when the expression of *SP5*/*btd* covers about 50% of the egg circumference, and before the ocular patches of *SP5*/*btd* expression have emerged. It is expressed as a sharply defined stripe; overlapping with the *SP5*/*btd* expression domain but shifted a few cell widths posteriorly (Fig. 3e, f). In the same way as *SP5*/*btd*, its expression becomes restricted to the head field as head condensation progresses (Fig. 3g). At the earliest stages of head condensation, the posterior limit of *col* expression lies posterior to the limit of *SP5*/*btd* expression, but a little later the *col* expression domain lies completely within the *SP5*/*btd* domain (Fig. 3h). *col* expression only spans the posterior part of this *SP5*/*btd* expression, in a domain that becomes the anterior intercalary and most posterior part of the antennal segment (Fig. 3h, i).

#### Subdivision of the *col* domain

By late stage 2.3, the *col* domain becomes subdivided into two stripes, one anterior and one posterior, connected laterally but separated medially by non-expressing cells (Fig. 3i). The more anterior *col* stripe lies at the posterior edge of antennal *SP5*/*btd* expression, and the posterior *col* stripe is located within the intercalary segmental region (Fig. 3i). Comparing both *col* and *en* to *SP5*/*btd* expression suggests that the anterior *col* stripe is coincident with antennal *en*, which marks the posterior compartment of the segment (see Fig. 1i). Finding *col* expression within this antennal region is surprising because no antennal *col* expression has been reported in insects or in the millipede *Glomeris* (see [22, 25–27]).

#### Fading of the antennal/intercalary *col* expression after head field condensation

Expression of *col* in this antennal/intercalary domain is transient. It fades rapidly after head condensation and transcripts have disappeared completely by early segmentation stages (stage 3.1, 3.2). Just after disappearance from this region, it will re-appear de novo in the anterior medial head region where it is a marker for neural cells [40]. We consider these two expression domains of *col* to be completely independent from one another. Additionally, at late stages of development *col* is expressed in a segmentally iterated pattern in the neuroectoderm and in a subset of mesodermal cells (not shown). The specific transient expression prior to the formation of the intercalary and antennal segments suggests a role of *col* in the formation or delimitation of these segments in the centipede.

#### *cap*‘*n*’*collar2* is expressed specifically in the mandibular segment

*Strigamia cnc2* is expressed specifically around the stomodaeum and more posteriorly within the mandibular segment, whereas *cnc1* is ubiquitously expressed in the embryo (see Additional file 1 and Additional file 4: Fig. S4 C, D and [33] for *Strigamia cnc* genes). The expression of *cnc2* in the mandibular segment is only clearly detectable at stage 3.2 (Fig. 3j, an early trunk segmentation stage, [34]) and subsequent stages (Fig. 3k). In *Drosophila* the anterior mandibular compartment of *cnc* expression overlaps and is positively regulated by *col* expression [24]. In *Strigamia col* does not extend into the mandibular territory at all. Its posterior border lies within the intercalary segment (see above). Also, *cnc2* is only expressed after *col* RNA has completely disappeared from its segmental expression domain. Hence, *col* expression and *cnc2* expression never overlap in the same cells and a direct regulatory action of *col* on *cnc2* (as found in the fly) can be excluded.

#### Emergence of the cephalic *hh* domain and first split

Two copies of the *hedgehog* (*hh*) gene are present in the *Strigamia* genome (*hh1* and *hh2*) [33]. They are not linked but show a high degree of sequence similarity. RNA-probes generated against the two orthologues showed the same pattern. Data presented here was generated using a probe against *hh1*.

**Fig. 4:**
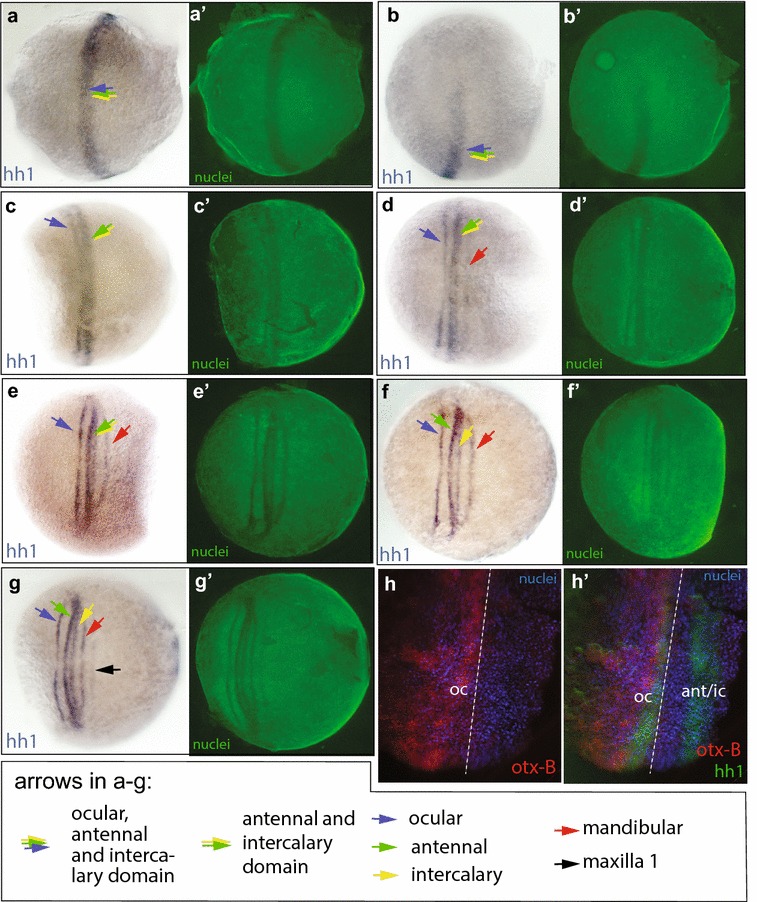
Expression of *hh1* (**a**–**g**) and *hh1* expression combined with *otx*-*B* (**h**). **a**, **c**–**g** ventral view. **b** lateral view. **a** (stage 2.2, early) First expression of *hh1* appears. **b** (same specimen as **a**) The early *hh1* domain covers about 50% of the egg circumference. **c** (stage 2.2, late) The 1st split of the early *hh* domain has begun laterally; this produces the ocular *hh stripe*. **d** (stage 2.2, late) The splitting-off of the ocular stripe has reached the medial domain. The mandibular stripe is faintly visible; it appears de novo, the lateral ends connecting to the initial, more anterior domain. **e** (stage 2.3, early) The ocular stripe has completely separated. The more posterior domain has begun to split into antennal and intercalary stripes. **f** (stage 2.3, early) Antennal and intercalary domains have split centrally but stay attached laterally. **g** (stage 2.3, early) First appearance of the 1st maxillary stripe. **h**/**h**′ (stage 2.2, late, showing the lateral part of the head domain). **h** *otx*-*B* channel only. **h**′ *otx*-*B* expression overlaps with the ocular *hh* stripe. Dashed line is in same place in **h** and **h**′ and marks the posterior limit of both the *otx*-*B* and the ocular *hh* domain. oc = ocular domain, ant/ic = antennal/intercalary domain

We have not been able to examine *hh* expression in the earliest blastoderm stage, but by early stage 2.2 *hh1* is expressed in one broad stripe covering about 50% of the egg circumference (Fig. 4a, b, stage comparable to Fig. 1b). This domain is in approximately the same position as the early stripe of *SP5*/*btd* expression, which later splits into ocular, antennal and intercalary expression domains.

From this single early *hh* domain, one narrow stripe splits off anteriorly, first laterally, and then medially (see Fig. 4c–e). It becomes the ocular *hh* stripe. Double stainings with *otx* show that the gap between the ocular *hh* stripe and the more posterior stripe of expression is coincident, or very nearly so, with the gap that appears between the early expression domains of *SP5*/*btd* and the *otx* genes (Fig. 2b, c′).

#### De novo appearance of mandibular *hh* expression

The more posterior part of the *hh* early expression domain initially remains as a single stripe in the antennal/intercalary region. Posterior to it mandibular expression appears (Fig. 4c). This mandibular expression most likely appears de novo. No ‘splitting-off’ event from the antennal/intercalary domain could be captured in the close series of developmental stages that were stained for *hh* expression.

#### Second split of the cephalic *hh* domain

Shortly after the appearance of mandibular *hh* expression, the antennal/intercalary *hh* domain splits into separate antennal and intercalary stripes (Fig. 4c, d). Antennal, intercalary and mandibular stripes of expression remain connected at their lateral ends (Fig. 4d–f), a pattern that is also seen for *en* expression (Fig. 1i). A similar split in the expression domains is also observed in the pattern of *SP5*/*btd* (see above), but this splitting is only completed at a slightly later stage in development (stage 3.1, Fig. 2f), possibly because of the greater A/P extent of the *SP5*/*btd* domains within each segment, which makes them directly adjacent or overlapping for longer.

### Summary of results

Among the studied factors *otx*-*B* and *SP5*/*btd* are expressed first, followed by *hh*, *col*, *en*, *ems* and *cnc2*. All early expression patterns reflect the head condensation process, whereby expression domains condense from a broad region of the early blastoderm to the ventral head field. Much of this movement presumably reflects the ventral, and to a limited extent posterior migration of cells in the anterior half of the egg to form the multi-layered head primordium. Condensation of the head territory does not follow a strict anterior to posterior progression: the region of the intercalary and antennal segment appears to condense first, followed by the ocular and then the most anterior medial region.

**Fig. 5:**
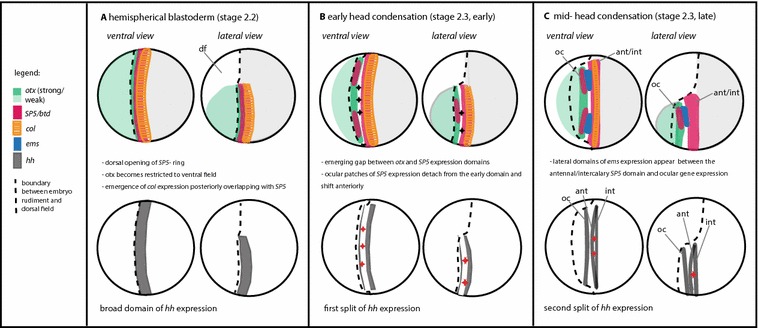
Schematic drawing of combined head gap gene expression (upper panel) in comparison with the dynamics of *hh1* expression (lower panel). The boundary between embryo rudiment and dorsal field, marked by a dashed line, is a gradual transition at the early stages, but becomes more defined during later development (compare Fig. 1). df = dorsal field (putatively ‘extra-embryonic’, characterised by lower cell density than the ventral part where the embryo forms) oc = ocular domain, ant/int = combined antennal and intercalary domain, ant = antennal domain, int = intercalary domain. Black arrows in **b** indicate split between the early *otx*-*B* and *SP5* domains, red arrows in **b** and **c** indicate splits of the *hh* domain

As the head condenses, initially abutting or overlapping domains of gene expression become more resolved, and some expression domains split, reflecting the formation of defined segments. These changes are summarised in Fig. 5. The upper diagrams illustrate the dynamics of early expression of head gap gene orthologues and *col*, whereas the lower diagrams show *hh* expression at the same phases of development.

## Discussion

### Head morphogenesis: shared characters in myriapods, spiders and sequentially segmenting insects

Symmetry breaking transitions from circumferential to ventrally restricted head gene expression occur in spiders and myriapods.

At the earliest stages of development in the myriapods *Glomeris* and *Strigamia*, early gene expression is often ring-like around the whole blastoderm, as we found for *SP5*/*btd* expression. As the embryonic primordium forms at the ventral side, these expression patterns become restricted to the embryo rudiment ([30, 42, 43] and this work).

Similarly, the early germ disc of the spider *P. tepidariorum* is radially symmetric. Anterior markers in the spider, such as *otd*-*1* and *hh*, are expressed in a complete ring around the early, radially symmetric germ disc [3], which is similar to the early hemispherical and ring-like expression of *SP5*/*btd* in *Strigamia*. After the radial symmetry of the germ disc in the spider is broken, the rings of expression open dorsally and *otd*-*1* and *hh* become restricted to stripes at the ventral side [3]. A similar process of ring opening is observed for *SP5*/*btd* in *Strigamia*. We are not sure whether *otx* and *hh* are also initially expressed in a complete ring—in the earliest embryos we have been able to stain, they cover only about 50% of the egg circumference, but we have successfully prepared very few embryos at these earliest stages, which are extremely difficult to handle.

In summary, a transition from circumferential gene expression to a ventral restriction is observed in both spiders and in myriapods. In spiders the migration of a cell cluster from the germ band centre to the rim, accompanied by *decapentaplegic* (*dpp*) signalling from these migrating cells, leads to a break of symmetry in the germ disc [44]. The trigger of the symmetry break in myriapods has so far not been identified, though a very early focus of *dpp* expressing cells has been observed in *Strigamia* [42].

#### The head rudiment only covers a ventral part of the anterior embryo in the centipede and in short-germ insects

The condensed head of *Strigamia* only covers a relatively small ventral part of the egg. Likewise in short-germ insects like the beetle *Tribolium* the head primordium does not cover the whole anterior part of the blastoderm but forms at the ventral side of the embryo, distant from the anterior pole of the egg, while dorsal and most anterior tissues are extra-embryonic (see [45, 46]). By contrast, in the long-germ insect *Drosophila*, the whole anterior cap of the embryo contributes to the head and only a small dorsal part of the anterior blastoderm forms amnioserosa [47]. Therefore, with respect to the ventral condensation process and the size of the head primordium relative to the egg-surface, head formation in *Strigamia* rather resembles the process in the sequentially segmenting insect *Tribolium* [20, 46] than head formation in the fly (see for example [1, 47]).

Long-germ development in insects is thought to have evolved multiple times independently from an ancestral short-germ type of development [48, 49], which is similar to the developmental mode in *Strigamia* where trunk segments are added sequentially from a posterior growth zone [34, 36, 50]. Hence, it is not surprising that also head formation in *Strigamia* is more similar to *Tribolium* than to *Drosophila*. Kittelmann et al. [46] suggested that the anterior displacement of the head anlagen, accompanied by the invention of the *bcd* dependent anterior patterning system, allowed for the evolution of long germ development in *Drosophila*.

#### The expression of head gap gene orthologues in *Strigamia* is more similar to that in the beetle *Tribolium* than either is to *Drosophila*

The head gap genes of *Drosophila* are characterised by staggered but extensively overlapping expression domains along the head anterior–posterior-axis: *otd* is expressed most anteriorly, followed by *ems* and then *btd* [6, 7, 10, 51].

In the beetle *Tribolium*, the relative sequence of *otd*, *ems* and *btd* expression is the same as in the fly, but the expression domains are more restricted and do not overlap to the large extent seen in *Drosophila* [20]. In the early *Tribolium* blastoderm, *otd*-*1* and *btd* are expressed in broad domains that are directly adjacent to one another: *otd*-*1* is expressed in two bilateral domains in the head lobes and *btd* is expressed directly posterior to it in a broad stripe. *ems* is not expressed at this early stage. Shortly thereafter, the *otd* and *btd* expression domains become separated by a gap, and within this gap the expression of *ems* emerges [20].

In the centipede *Strigamia*, the dynamics of early head gap gene expression are very similar to those in *Tribolium*: at the early blastoderm stage *otx* is expressed in the anterior head and *SP5*/*btd* is expressed directly posterior to it. At the interface, a few cells express both *otx*/*otd* and *SP5*/*btd* in *Strigamia*, as well as in the beetle [20]. With condensation of the head field, *otx*/*otd* and *SP5*/*btd* expression become separated in *Strigamia* as they do in *Tribolium*, and in both species *ems* expression emerges between them in two lateral domains (compare schematic drawing in Fig. 5 and [20]).

During subsequent development, the resolution of these domains to specific segments differs in *Tribolium* and *Strigamia*. Whereas in *Strigamia* the early *SP5*/*btd* domain divides into ocular patches and an antennal/intercalary domain, thus marking all cephalic segments, the early *SP5*/*btd* domain in *Tribolium* shrinks to become positioned in the mandibular segment [20]. In addition, early *ems* expression in *Strigamia* overlaps in its posterior part with antennal *SP5*/*btd* expression, but there is no overlap of *SP5*/*btd* and *ems* in *Tribolium*. Hence, the later expression of *SP5*/*btd* is positioned relatively more posterior in *Tribolium* than in *Strigamia*, although in both organisms the expression domain is directly posterior to *otd* at early development, and in both, *otd*/*otx* marks the ocular segment during later development.

In *Strigamia*, we never see the large areas of overlap that characterise early head gap gene expression in *Drosophila*. In *Drosophila*, *otd* is expressed in a domain that covers the ocular and the whole antennal segment (see [1]), whereas in *Strigamia* the posterior limit of *otx* expression is the ocular segment. The early head stripe of *btd* expression in *Drosophila* covers the antennal, intercalary and mandibular segment [1] but in *Strigamia* the posterior limit of early expression of *SP5*/*btd* is more anterior, in the intercalary segment. In *Drosophila*, *ems* is expressed in a large domain covering the antennal and intercalary segment [1]. In *Strigamia*, the earliest expression of *ems* only covers a small territory in the anterior antennal segment. This expression initiates late and seems to be part of a pattern of metameric expression that appears in every following segment in anterior to posterior progression.

In conclusion, there are similarities in head gap gene expression between *Drosophila*, *Tribolium* and *Strigamia*, in the AP-order of expression domains, in the early expression of *otx* and *SP5*/*btd*, and to some extent in the dynamics of *SP5*/*btd* pattern resolution. However, the overlap of early gap gene expression domains, and the extensive early expression of *ems* in *Drosophila* head patterning, are not found in the centipede, or in *Tribolium*.

### Implications of gap gene expression in *Strigamia*

The *Drosophila* head gap genes are crucial for correct patterning of head segments: loss of any of these three genes results in the deletion of several contiguous segments in *Tribolium*, only *otd* has a severe head phenotype when knocked down via RNAi: extreme phenotypes delete all head and gnathal segments, and may also affect the thorax [19, 20]. In contrast, RNAi mediated knockdown of *btd* (*SP5*) did not lead to any head phenotype, and the knockdown of *ems* only affected the head cuticle of the antennal segment: in the strongest *ems* RNAi phenotypes, the ocular and antennal *wingless* (*wg*) stripes were merged. These limited phenotypes may reflect limitations of the RNAi gene knockdown, but that seems unlikely, given the general efficacy of RNAi in *Tribolium*.

The lack of an *SP5*/*btd* phenotype in *Tribolium* is an unexpected finding considering the deep conservation of early head specific *SP5*/*btd* expression in myriapods and insects. It is hard to believe that the highly conserved domain of anterior *SP5*/*btd* expression lacks function in most systems other than the fly. A re-investigation of *SP5*/*btd* function in other arthropod models, and possible functional redundancy with the *SP6*-*9* orthologues, the expression of which overlaps with *SP5*/*btd* in the beetle [39], as it does in the centipede, might shed light on the evolution of the functional role of these *SP* genes.

*Tribolium otd* also has a characterised role in dorsal–ventral patterning, which by itself might explain the anterior defects after *otd* knockdown [45]. However, given the widely conserved early anterior expression of *otx* genes across animals [41, 52], it seems likely that *otx* genes in the centipede function in the correct establishment of the anterior head, and specifically in the ocular region.

### Conclusions: shared patterns of head gap gene domains in beetle and centipede and their alterations in the fly

The striking similarities between *Tribolium* and *Strigamia* in the early *otd*-*ems*-*btd* pattern suggest that this may be an ancestral aspect of arthropod patterning. The observation that broad gap gene-like expression of *ems* is found neither in the centipede nor in the beetle suggests that *ems* has only been recruited to a major role in regional anterior patterning in the lineage leading to the fly. This view is supported by functional data from *Tribolium*, where *ems* knockdown only leads to subtle effects on antennal development [20].

The use of extensive, overlapping domains of head gap gene expression to pattern anterior segments can hence be interpreted as an invention of the Diptera, where it is associated with the long germ type of development, and might contribute to the fast development of the fly. The long-germ development by itself is an example where the fly has evolved a divergent mechanism that facilitates fast development: each trunk segment is specified by a unique combinatorial input of gap genes [53], whereas in other arthropods segments are added sequentially from a posterior growth zone (e.g. [36, 54–56]. A classical gap gene patterning system, where early expressed genes define broad regions of the embryo and directly or indirectly control segmentation genes has, however, not been described outside the insects [49].

### *col* and *cnc* patterning

The gene network surrounding the intercalary segment, where *col* acts at an intermediate level between the early expressed head gap genes and segment polarity gene expression, is probably the best understood part of the *Drosophila* head segmentation mechanism [22, 27, 57]. We compared this gene network to the expression data from *Strigamia*.

#### An anterior shift of centipede *col* expression by half a segment in comparison with insects

Early segmental *col* expression in the insects, and also in the millipede *Glomeris*, is mainly localised within the intercalary segment [25–27]. In both *Drosophila* and *Tribolium*, the posterior limit of expression extends into the mandibular segment and the anterior limit in all species previously examined lies within the intercalary segment [22, 25, 26, 58]. In the fly *col* expression is also dynamic: it is first expressed more widely in the anterior mandibular and posterior intercalary segment and then becomes restricted to the posterior intercalary segment only. The late *col* domain in *Drosophila* includes the intercalary spots of *en* expression and also tissue posterior to that, but does not overlap with antennal *en* expression [22]. Hence, *col* expression is about half a segment more posterior in *Drosophila* than it is in *Strigamia*, where the anterior limit is in the antennal segment. Irrespective of the slightly different position of the expression domains, in both the fly and in the centipede the coherent domain later splits into two stripes that are connected at the lateral ends [22].

In the millipede, the anterior limit of *col* expression is also clearly distant from antennal *en* expression. The precise posterior extent of the expression remains unclear; however, as in the insects expression does not extend anteriorly into the antennal segment [25]. The work by Janssen et al. [25] also shows *col* expression in the centipede *Lithobius forficatus*. Unfortunately, these data are presented without a segmental marker as counterstain, so it is not possible to locate the *col* domain precisely, or to assess whether *col* expression is shifted anteriorly in this other centipede, as it is in *Strigamia*.

#### *col* as a putative mediator between head gap gene expression and segment polarity gene expression

In *Drosophila col* has been characterised as a mediator through which the head gap gene *btd* activates *hh* and *en* expression within the intercalary segment [22]. Here, the *col* domain lies completely within the *btd* domain, and in *btd* mutant embryos *col* expression is completely lost [27]. In *Strigamia* early expression of *SP5*/*btd* and *col* co-localise to a large degree and a regulatory input of *SP5*/*btd* on *col* seems possible. *Strigamia col* may also be involved in activating segment polarity gene expression within its intercalary expression domain, as does *col* in *Drosophila* [22]. A regulatory input of *ems* on *col*, as found in the fly [22] is, however, not possible in *Strigamia* as the two factors are not co-expressed.

In the posterior part of the *Drosophila* blastoderm *col* is repressed by *eve*; in an *eve* mutant embryo an additional *col* stripe appears posterior to the intercalary one [22]. A similar down-regulation of *eve1* by *col* is possible in *Strigamia*: during early blastoderm *eve1* characterises the tissue that will give rise to the mandibular and posteriorly following segments, an area where *col* is not expressed.

#### Separation of the *cnc2* and *col* expression domains in *Strigamia*

*cnc* orthologues are expressed within the labrum and the mandibular segment of myriapods and pancrustaceans [30, 32, 58]. In *Drosophila*, *cnc* in the mandibular segment depends on the early head domain of *btd* [59]. By contrast, the early head expression of *Strigamia SP5*/*btd* does not co-localise with mandibular *cnc2* expression. The later segmental expression of *SP5*/*btd*, which includes the mandibular segment, is unlikely to activate *cnc2* because *SP5*/*btd* is restricted to the central part of the segment, whereas *cnc2* fills the complete segmental area.

*cnc* expression in the fly also depends on *col* in the posterior intercalary segment [22]. By contrast, *Strigamia col* expression is not only spatially but also temporally separated from *cnc2* expression. Hence, a direct activation of *cnc2* by *col* can be excluded, and in contrast to *Drosophila*, *cnc2* activation in *Strigamia* is independent of *col* and also of *SP5*/*btd* function. Interestingly, also in the beetle, *cnc* expression in the mandibular segment is not altered in *col* knockdown embryos [26].

In conclusion, some of the dynamics of the combined head gap gene, *col*, *cnc2* and segmentation gene expressions in *Drosophila* are similar to those in *Strigamia*, which suggests a partial conservation of gene interactions between insects and myriapods. But there are also clear differences, as for example the anterior shift of *col* expression in *Strigamia* and the late expression of *cnc2*, separated from the *SP5*/*btd* and *col* expression domains.

### *hedgehog* domain splitting

Gene expression patterns during early segmentation of the head field of *Strigamia* show that the ocular, antennal and intercalary segments derive from a shared domain, which is specified at the blastoderm stage. This is strikingly shown by the pattern of early *hedgehog* (*hh*) expression: one initial domain of expression splits twice to give rise to the ocular, antennal and intercalary *hh* stripes. At about the same time, or shortly afterwards, a single *engrailed* (*en*) stripe splits to give rise to the antennal, intercalary and mandibular *en* stripes, presumably as a downstream consequence of the earlier *hh* patterning. In the only other myriapod where head segmentation has been examined, the millipede *Glomeris*, the ocular and antennal stripe of *hh* expression also split from a shared domain. The intercalary stripe by contrast is described as appearing de novo after some delay [29].

The dynamic of *hh* stripe splitting in the spider *P. tepidariorum* is slightly different. An early broad domain of *hh* expression in the anterior part of the germ band undergoes two splitting events, as in *Strigamia*, but the first split gives rise to a posterior stripe, which becomes the pedipalpal *hh* stripe, and an anterior stripe that undergoes a second round of splitting to give rise to the most anterior (cephalic) stripe of *hh* expression and to the cheliceral stripe posterior to it [3, 4].

Current understanding of spider head segments, based on *hox* gene expression, homologises the spider cheliceral segment with the insect (and myriapod) antennal segment, and the pedipalpal with the intercalary segment [60, 61]. Assuming that the cephalic *hh* domain corresponds to ocular *hh* expression in myriapods, the early *hh* expression domain comprises the same three respective segments. However, the order of stripe splitting differs between the spider and the centipede: in the spider it is the anterior of the first two stripes that splits again [4], whereas in *Strigamia* it is the posterior stripe that undergoes a second split.

The subdivision of an early *hh* domain into three cephalic segmental stripes has also been described in *Drosophila*. In the fly, a wedge-shaped domain of *hh* expression is established at blastoderm stage in the cephalic region of the embryo. During gastrulation and germ band extension, this domain subdivides into an intercalary, an antennal and a procephalic (ocular) stripe [28]. Unfortunately, this early observation has not been followed up by more recent studies with current techniques. However, the fact that *hh* stripe splitting occurs in centipedes, millipedes, spiders and potentially also in insects suggests that this process may be a basic principle of head segmentation in arthropods.

In the spider, Kanayama et al. [4] have shown that the initial domain of *hh* expression is dynamic with respect to the cell population. It moves from the most anterior rim of the germ disc to a more posterior position, where it then splits into the cephalic segmental stripes. The initial domain overlaps with the posterior edge of *otd* expression, which is similarly dynamic. RNAi mediated knockdown of spider *otd* caused *hh* expression to remain static at the germ band rim. Cell-specific knockdown of *otd* after the *hh* travelling phase led to an interruption of the stripe splitting events through loss of *hh* in the *otd* negative cells. Hence, *otd* in the spider is required to maintain *hh* expression during both the travelling and the splitting phase of early *hh* expression [4].

We have no evidence for expression of *hh* that travels over cells before the onset of splitting during early *Strigamia* development, although such a process cannot be excluded based on the methods used here. As in the spider, at the onset of its subdivision, the initial *hh* domain is located some way from the anterior rim of the condensed head field. We showed that *Strigamia otx* gene expression is overlapping with the anterior part of this broad initial *hh* domain. The most posterior *otx* expression is then found in the ocular segment, where it overlaps with the most anterior stripe of *hh*. The more posterior, antennal/intercalary *hh* domain, which splits again, does not overlap with *otd*. Thus, in *Strigamia*, *otx* expression might be required for maintenance of *hh* in its ocular domain, but it is certainly not required for maintaining *hh* expression during the second stripe splitting event.

## Conclusions

The head patterning system in the centipede, as inferred from our analysis of gene expression, reveals some remarkable features.

A set of gap gene orthologous genes is expressed during early head patterning in *Strigamia*, *Tribolium* and *Drosophila*. They are expressed in the same relative anterior/posterior order, but expression domains in *Drosophila* are broader, with larger areas of overlap [1], which we interpret as part of a patterning mechanism that is specific to the fly lineage. Greater similarities exist between the early dynamics of gene expression in *Strigamia* and the sequentially segmenting insect *Tribolium* [20].

The downstream network in the intercalary and the mandibular segment involves at least some of the same factors in insects and myriapods. Our analysis of the spatiotemporal dynamics of gene expression suggests that some but not all gene interactions are conserved. This raises a question as to head patterning in crustaceans, as current phylogenies place insects as an in-group of crustaceans [62–64]. Crustacean head segments differ from those of insects and myriapods as they have a second antennal segment instead of an appendage-less intercalary segment. *col*-dependent patterning seems to be absent from this second antennal segment [26], whereas expression of *cnc* in the mandibular segment of crustaceans is conserved [32].

Finally, a mode of head segmentation involving the splitting of *hh* domains, which appears quite different from segmentation in the trunk, may be a basic principle of arthropod head development. Future studies should address the molecular mechanism of this segmentation mode in different species, and its interdependence with tissue mechanics.

## Methods

### Embryo collection and fixation

*Strigamia maritima* embryos were collected from a wild population near Brora, Scotland [50]. The material was fixed for several days in 4% formaldehyde/0.5 × PBS and then transferred to methanol for storage at – 20 °C. Embryos were staged according to morphological features (according to [34]).

### Genetic resources and gene classification

Genomic resources for *S. maritima* are available at http://www.ncbi.nlm.nih.gov/assembly/322118/ and a manually annotated version of the genome is available at http://metazoa.ensembl.org/Strigamia_maritima/Info/Index.

Gene orthologues were identified by Blast searches and reciprocal testing. A list of the identified genes with their Ensembl gene IDs is given in Additional file 1: Table S1. The phylogenetic tree for the classification of SP genes (Additional file 2: Fig. S1 and Additional file 3: Fig. S2) was created using Phylemon2 [65]. Multiple sequence alignments were performed on protein sequences using MUSCLE [66], and the gene tree was built based on maximum likelihood analysis in PhyML [67]. Tree calculation parameters are given in the legend of Additional file 2: Fig. S1.

### Gene amplification and RNA in situ staining of gene expression

Specific primers were designed against the identified gene sequences. Products were amplified by standard PCR and subsequently cloned into the pGEM-T-Easy vector system (Promega). Inserts were verified by sequencing and then used as templates for in situ probe synthesis. Single and double colorimetric in situs using a BM-purple and a fast red staining reaction were performed as described in [35, 50]. All embryos were counterstained with the nuclear dye Hoechst (H34580 2 µg/ml) or Sytox green (1 µM).

### Image acquisition

After the staining procedure, specimens were immersed in 90% Glycerol for microscopy. Whole mount in situ stained embryos were photographed using a Leica MZFLIII stereomicroscope with attached DFC500 camera or a Zeiss Axiophot compound microscope with attached Leica DFC300FX camera. Picture stacks with different focal planes were taken of the spherical shaped embryos and then reconstructed to images of whole embryos using the software Helicon Focus (Helicon soft Ltd.). In addition, photographs of the nuclear stain were always taken from the same angle as a reference for stage and morphology of the embryo. For the overlay of fluorescence pictures (Hoechst, Fast Red) with bright-field pictures of the BM-purple stain an RGB picture was created using Adobe Photoshop. The Fast Red stain was assigned to the red channel, the Hoechst stain to the blue channel and the BM-purple stain to the green channel. The latter was colour inverted to change from a dark stain on a white background to a light stain on dark background, which then allowed creating the overlay with the two other channels. Some of the embryos were imaged using a Leica Sp5 confocal microscope using the 543 mm laser line for fast red visualisation and the 405 mm diode for the Hoechst stain. Brightness and contrast of whole images were adjusted using Photoshop CS5.

## Additional files

**Additional file 1.** Genomic organisation and additional expression patterns of centipede head patterning gene orthologues.

**Additional file 2: Fig. S1.** Maximum likelihood gene tree of arthropod, cnidarian, mouse, zebrafish and human SP factors, unrooted. Tree based on “muscle” protein alignment of the conserved zinc finger region. An *SP6-9* clade (only to the exclusion of vertebrate and cnidarian *SP6* genes) has good bootstrap support (76/100). Within this clade *Strigamia SP6-9* (SMAR004954) clusters with the remaining arthropod *SP6-9* genes. All SP1-4 proteins, including *Strigamia* SP1-4 (SMAR004952) group together; this clade has 65/100 bootstrap support. *Strigamia SP5* (SMAR004861) forms a clade with other *SP5* and *btd* genes, only to the exclusion of *Folsomia SP5*. The SP5 group is not statistically robust though (27/100). Mm=*Mus musculus* (*Mm_SP1*: NP_038700.2; *Mm_SP2*: CAM21905.1; *Mm_SP3*; AAX90615.1; *Mm_SP4*: NP_033265.3; *Mm_SP5*: NP_071880.1; *Mm_SP6*: NP_112460.1; *Mm_SP7*: NP_569725.1; *Mm_SP8*: NP_796056.2; *Mm_SP9*: NP_001005343.1), Hs=*Homo sapiens* (*Hs_SP1*: NP_612482.2; *Hs_SP2*: NP_003101.3; *Hs_SP3*: NP_003102.1; *Hs_SP4*: NP_003103.2; *Hs_SP5*: NP_001003845.1; *Hs_SP6*: NP_954871.1; *Hs_SP7*: NP_690599.1; *Hs_SP8*: NP_874359.2; *Hs_SP9*: NP_001138722.1), Dr=*Danio rerio* (*Dr_SP1*: NP_997827.1; *Dr_SP2*: NP_001093452.1; *Dr_SP3*: NP_001082967.1; *Dr_SP4*: NP_956418.1; *Dr_SP5*: NP_851304.1; *Dr_SP6*: NP_991195.1; *Dr_SP7*: NP_998028.1; *Dr_SP8*: NP_991113.1; *Dr_SP9*: NP_998125.2), Dm=*Drosophila melanogaster* (*Dm_CG5669*: AAF56261.1; *Dm_btd*: NP_511100.1, *Dm_Sp69*: NP_572579.2), Fc=*Folsomia candida* (*Fc_SP14*: CBH30974.1; *Fc_Sp5*: FN562986; *Fc_Sp6-9*: FN562987), Tc=*Tribolium castaneum* (*Tc_SP1-4*: XP_972252.1; *Tc_btd*: NP_001107792.1; *Tc_Sp-like*: NP_001034509.1), Nv=*Nematostella vectensis* (*Nv_SP1-4*: XP_001635004.1; *Nv_SP5*: XP_001635002.1; *Nv_SP6-9*: XP_001634948.1), Gm=*Glomeris marginata* (*Gm_btdI*: CAK50835.1), Ph=*Parhyale hawaiensis* (*Ph_Sp14*: CBH30980.1; *Ph_Sp6-9*: FN562992.1).

**Additional file 3: Fig. S2.** Genomic organisation and protein structure of head patterning genes. **A** Protein structure of the three centipede SP factors. The SP box motif is missing in the short SP5 protein. **B** Conserved centipede SP gene cluster. **C** Close linkage of *otx-A* and *otx-B*, and conserved microsynteny with an EH-domain-binding protein 1. *otx-C* maps onto a different genomic scaffold.

**Additional file 4: Fig. S3.** Expression of *SP6-9* (A-D) and *SP1-4* (E-H) during early *Strigamia* development. A-F, H: ventral views, G: lateral view. **A** (stage 2.2, early) *SP6-9* is expressed in the posterior hemisphere of the embryo. **B** (stage 2.2 late) *SP6-9* is expressed in anterior segmental domains where is overlaps with *SP5* expression (compare figure 2) and in a broad domain at the posterior pole. **C** (stage 2.3) and **D** (stage 3.1) Segmental expression of *SP6-9* and expression within the posterior growth zone. **E** (stage 2.2, early) *SP1-4* is uniformly expressed during blastoderm stage, a seemingly stronger expression in the posterior is due to the higher cell density in this area. **F** and **G** (stage 2.3, late; G is a lateral view of same specimen). Expression is uniform, darker areas are correlated with the germ band and with areas of multi-layered tissue/high cell density. **H** (stage 4.3) *SP1-4* expression at a mid-segmentation stage. Expression is stronger in the germ band than in the extra-embryonic territory, but largely reflects the morphology. **oc**= ocular domain, **ant/int**= antennal and intercalary expression, **md**= mandibular domain, **mx1**= 1^st^ maxillary domain.

**Additional file 5: Fig. S4.** Expression of *otx-B* (single stains) and *cnc1*. All in ventral view. **A** (stage 2.2, early) *otx-B* is expressed in an anterior cap and more strongly at the posterior margin of its expression domain. **B** (stage 2.3) *otx-B* expression is strong in an ocular domain and weaker in the prospective head field anterior to that. Expression is also seen along the midline. **C** (stage 2.3, late) and **D** (stage 4.1) *cnc1* is expressed in a ubiquitous pattern, darker staining in areas of dense tissue. **oc**= ocular domain, **ml**= midline.

## References

[1] Cohen S, Jürgens G (1991). *Drosophila* headlines. Trends Genet.

[2] Peel A (2004). The evolution of arthropod segmentation mechanisms. BioEssays.

[3] Pechmann M, McGregor A, Schwager E, Feitosa N, Damen W (2009). Dynamic gene expression is required for anterior regionalization in a spider. Proc Natl Acad Sci USA.

[4] Kanayama M, Akiyama-Oda Y, Nishimura O, Tarui H, Agata K, Oda H (2011). Travelling and splitting of a wave of hedgehog expression involved in spider-head segmentation. Nat Commun.

[5] St Johnston D, Nüsslein-Volhard C (1992). The origin of pattern and polarity in the *Drosophila* embryo. Cell.

[6] Cohen S, Juergens G (1990). Mediation of *Drosophila* head development by gap-like segmentation genes. Nature.

[7] Finkelstein R, Perrimon N (1990). The *orthodenticle* gene is regulated by bicoid and torso and specifies *Drosophila* head development. Nature.

[8] Walldorf U, Gehring WJ (1992). *Empty spiracles*, a gap gene containing a homeobox involved in *Drosophila* head development. EMBO J.

[9] Wimmer EA, Simpson-Brose M, Cohen SM, Desplan C, Jäckle H (1995). Trans- and cis-acting requirement for blastodermal expression of the head gap gene *buttonhead*. Mech Dev.

[10] Dalton D, Chadwick R, McGinnis W (1989). Expression and embryonic function of *empty spiracles*: a *Drosophila* homeobox gene with two patterning functions on the anterior-posterior axis of the embryo. Genes Dev.

[11] Grossniklaus U, Cadigan KM, Gehring WJ (1994). Three maternal coordinate systems cooperate in the patterning of the *Drosophila* head. Development.

[12] Mohler J (1995). Spatial regulation of segment polarity gene expression in the anterior terminal region of the *Drosophila* blastoderm embryo. Mech Dev.

[13] Akam M, Averof M, Castelli-Gair J, Daw R, Falciani F, Ferrier D. The evolving role of Hox genes in arthropods. Development 1994;Supplement:209–15. 7579521

[14] Stauber M, Jäckle H, Schmidt-Ott U (1999). The anterior determinant *bicoid* of *Drosophila* is a derived *Hox* class 3 gene. Proc Natl Acad Sci USA.

[15] Wotton KR, Jimenez-Guri E, Jaeger J (2015). Maternal co-ordinate gene regulation and axis polarity in the scuttle fly *Megaselia abdita*. PLoS Genet.

[16] Fu J, Posnien N, Bolognesi R, Fischer T, Rayl P, Oberhofer G, Kitzmann P, Brown S, Bucher G (2012). Asymmetrically expressed axin required for anterior development in *Tribolium*. Proc Natl Acad Sci.

[17] Klomp J, Athy D, Kwan CW, Bloch NI, Sandmann T, Lemke S, Schmidt-Ott U (2015). A cysteine-clamp gene drives embryo polarity in the midge *Chironomus*. Science.

[18] Li Y, Brown SJ, Hausdorf B, Tautz D, Denell RE, Finkelstein R (1996). Two *orthodenticle*-related genes in the short-germ beetle *Tribolium castaneum*. Dev Genes Evol.

[19] Schröder R (2003). The genes *orthodenticle* and *hunchback* substitute for *bicoid* in the beetle *Tribolium*. Nature.

[20] Schinko JB, Kreuzer N, Offen N, Posnien N, Wimmer EA, Bucher G (2008). Divergent functions of *orthodenticle*, *empty spiracles* and *buttonhead* in early head patterning of the beetle *Tribolium castaneum* (Coleoptera). Dev Biol.

[21] Birkan M, Schaeper ND, Chipman AD (2011). Early patterning and blastodermal fate map of the head in the milkweed bug *Oncopeltus fasciatus*. Evol Dev.

[22] Crozatier M, Valle D, Dubois L, Ibnsouda S, Vincent A (1999). Head versus trunk patterning in the *Drosophila* embryo; *collier* requirement for formation of the intercalary segment. Development.

[23] Ntini E, Wimmer EA (2011). Second order regulator Collier directly controls intercalary-specific segment polarity gene expression. Dev Biol.

[24] Mohler J, Mahaffey JW, Deutsch E, Vani K (1995). Control of *Drosophila* head segment identity by the bZIP homeotic gene *cnc*. Development.

[25] Janssen R, Damen WG, Budd GE (2011). Expression of *collier* in the premandibular segment of myriapods: support for the traditional Atelocerata concept or a case of convergence?. BMC Evol Biol.

[26] Schaeper N, Pechmann M, Damen W, Prpic N, Wimmer EA (2010). Evolutionary plasticity of collier function in head development of diverse arthropods. Dev Biol.

[27] Crozatier M, Valle D, Dubois L, Ibnsouda S, Vincent A (1996). *Collier*, a novel regulator of Drosophila head development, is expressed in a single mitotic domain. Curr Biol.

[28] Lee JJ, Von Kessler DP, Parks S, Beachy PA (1992). Secretion and localized transcription suggest a role in positional signaling for products of the segmentation gene *hedgehog*. Cell.

[29] Janssen R (2012). Segment polarity gene expression in a myriapod reveals conserved and diverged aspects of early head patterning in arthropods. Dev Genes Evol.

[30] Janssen R, Budd GE, Damen WGM (2011). Gene expression suggests conserved mechanisms patterning the heads of insects and myriapods. Dev Biol.

[31] Browne WE, Schmid BGM, Wimmer EA, Martindale MQ (2006). Expression of *otd* orthologs in the amphipod crustacean, *Parhyale hawaiensis*. Dev Genes Evol.

[32] Sharma PP, Gupta T, Schwager EE, Wheeler WC, Extavour CG. Subdivision of arthropod *cap*-*n*-*collar* expression domains is restricted to Mandibulata. EvoDevo. 2014;5. doi:10.1186/2041-9139-5-3. 10.1186/2041-9139-5-3 PMC3897911 24405788

[33] Chipman AD, Ferrier DEK, Brena C, Qu J, Hughes DST, Schröder R, Torres-Oliva M, Znassi N, Jiang H, Almeida FC, Alonso CR, Apostolou Z, Aqrawi P, Arthur W, Barna JCJ, Blankenburg KP, Brites D, Capella-Gutiérrez S, Coyle M, Dearden PK, Du Pasquier L, Duncan EJ, Ebert D, Eibner C, Erikson G, Evans PD, Extavour CG, Francisco L, Gabaldón T, Gillis WJ (2014). The first myriapod genome sequence reveals conservative arthropod gene content and genome organisation in the centipede *Strigamia maritima*. PLoS Biol.

[34] Brena C, Akam M (2012). The embryonic development of the centipede *Strigamia maritima*. Dev Biol.

[35] Chipman AD, Stollewerk A (2006). Specification of neural precursor identity in the geophilomorph centipede *Strigamia maritima*. Dev Biol.

[36] Chipman AD, Akam M (2008). The segmentation cascade in the centipede *Strigamia maritima*: involvement of the Notch pathway and pair-rule gene homologues. Dev Biol.

[37] Brena C, Akam M (2013). An analysis of segmentation dynamics throughout embryogenesis in the centipede *Strigamia maritima*. BMC Biol.

[38] Green J, Akam M (2013). Evolution of the pair rule gene network: insights from a centipede. Dev Biol.

[39] Schaeper ND, Prpic N-M, Wimmer EA (2010). A clustered set of three Sp-family genes is ancestral in the Metazoa: evidence from sequence analysis, protein domain structure, developmental expression patterns and chromosomal location. BMC Evol Biol.

[40] Hunnekuhl VS, Akam M (2014). An anterior medial cell population with an apical-organ-like transcriptional profile that pioneers the central nervous system in the centipede *Strigamia maritima*. Dev Biol.

[41] Steinmetz P, Urbach R, Posnien N, Eriksson J, Kostyuchenko R, Brena C, Guy K, Akam M, Bucher G, Arendt D (2010). *Six3* demarcates the anterior-most developing brain region in bilaterian animals. Evodevo.

[42] Brena C. Myriapoda. In: Wanninger A, editors. Evolutionary developmental biology of invertebrates 3: Ecdysozoa I: non-tetraconata. Vienna: Springer; 2015. p. 141–89.

[43] Janssen R, Damen WGM, Budd GE (2012). Expression of pair rule gene orthologs in the blastoderm of a myriapod: evidence for pair rule-like mechanisms?. BMC Dev Biol.

[44] Akiyama-Oda Y, Oda H (2010). Cell migration that orients the dorsoventral axis is coordinated with anteroposterior patterning mediated by Hedgehog signaling in the early spider embryo. Development.

[45] Kotkamp K, Klingler M, Schoppmeier M (2010). Apparent role of *Tribolium orthodenticle* in anteroposterior blastoderm patterning largely reflects novel functions in dorsoventral axis formation and cell survival. Development.

[46] Kittelmann S, Posnien N, Bucher G (2012). Changes in anterior head patterning underlie the evolution of long germ embryogenesis. Dev Biol.

[47] Hartenstein V, Technau GM, Campos-Ortega JA (1985). Fate-mapping in wild-type *Drosophila melanogaster* III. A fate map of the blastoderm. *Roux’s Arch*. Dev Biol.

[48] Davis GK, Patel NH (2002). Short, long, and beyond: molecular and embryological approaches to insect segmentation. Annu Rev Entomol.

[49] Jaeger J (2011). The gap gene network. Cell Mol Life Sci.

[50] Chipman AD, Arthur W, Akam M (2004). Early development and segment formation in the centipede, *Strigamia maritima* (Geophilomorpha). Evol Dev.

[51] Wimmer EA, Cohen SM, Jäckle H, Desplan C (1997). *Buttonhead* does not contribute to a combinatorial code proposed for *Drosophila* head development. Development.

[52] Hirth F, Reichert H (1999). Conserved genetic programs in insect and mammalian brain development. BioEssays.

[53] Akam M (1989). Making stripes inelegantly. Nature.

[54] Stollewerk A, Schoppmeier M, Damen W (2003). Involvement of Notch and Delta genes in spider segmentation. Nature.

[55] Pueyo JI, Lanfear R, Couso JP (2008). Ancestral Notch-mediated segmentation revealed in the cockroach *Periplaneta americana*. Proc Natl Acad Sci USA.

[56] Sarrazin AF, Peel AD, Averof M (2012). A segmentation clock with two-segment periodicity in insects. Science.

[57] Seecoomar M, Agarwal S, Vani K, Yang G, Mohler J (2000). *knot* is required for the hypopharyngeal lobe and its derivatives in the *Drosophila* embryo. Mech Dev.

[58] Economou A, Telford M (2009). Comparative gene expression in the heads of *Drosophila melanogaster* and *Tribolium castaneum* and the segmental affinity of the *Drosophila* hypopharyngeal lobes. Evol Dev.

[59] Mohler J (1993). Genetic regulation of CNC expression in the pharnygeal primordia of *Drosophila* blastoderm embryos. Dev Biol.

[60] Damen WG, Hausdorf M, Seyfarth EA, Tautz D (1998). A conserved mode of head segmentation in arthropods revealed by the expression pattern of Hox genes in a spider. Proc Natl Acad Sci USA.

[61] Telford MJ, Thomas RH (1998). Expression of homeobox genes shows chelicerate arthropods retain their deutocerebral segment. Proc Natl Acad Sci USA.

[62] Regier JC, Shultz JW (1997). Molecular phylogeny of the major arthropod groups indicates polyphyly of crustaceans and a new hypothesis for the origin of hexapods. Mol Biol Evol.

[63] Regier JC, Shultz JW, Kambic RE (2005). Pancrustacean phylogeny: hexapods are terrestrial crustaceans and maxillopods are not monophyletic. Proc Biol Sci.

[64] Regier JC, Shultz JW, Zwick A, Hussey A, Ball B, Wetzer R, Martin JW, Cunningham CW (2010). Arthropod relationships revealed by phylogenomic analysis of nuclear protein-coding sequences. Nature.

[65] Sánchez R, Serra F, Tárraga J, Medina I, Carbonell J, Pulido L, de María A, Capella-Gutíerrez S, Huerta-Cepas J, Gabaldón T, Dopazo J, Dopazo H (2011). Phylemon 2.0: a suite of web-tools for molecular evolution, phylogenetics, phylogenomics and hypotheses testing. Nucleic Acids Res.

[66] Edgar RC (2004). MUSCLE: a multiple sequence alignment method with reduced time and space complexity. BMC Bioinform.

[67] Guindon S, Gascuel O (2003). A simple, fast, and accurate algorithm to estimate large phylogenies by maximum likelihood. Syst Biol.

