# Supplementary material for: Formation and subdivision of the head field in the centipede Strigamia maritima, as revealed by the expression of head gap gene orthologues and hedgehog dynamics
Source: EvoDevo. 2017 Oct 23;8:18. doi: 10.1186/s13227-017-0082-x (PMC5654096; doi:10.1186/s13227-017-0082-x)
Supplement: Supplementary file 1 — Additional file 1. Genomic organisation and additional expression patterns of centipede head patterning gene orthologues. [file 13227_2017_82_MOESM1_ESM.docx]

**Supplementary material**

**1 Genomic organisation and additional expression patterns of centipede head patterning gene orthologues**

***1.1 The Strigamia SP5/buttonhead gene is part of a presumably intact SP family gene cluster***

Invertebrate *SP* genes group into three different clades: *SP1-4*, *SP5* and *SP6-9*, named after their mouse and human evolutionary homologues of the expanded vertebrate gene family [1, 2]. It was suggested by Schaeper *et al*. [2] that a cluster of three genes that are linked and show synteny with the Hox cluster is ancestral to eumetazoans. Such a clustering of three *SP* genes and chromosomal location in proximity to the Hox genes is also predicted by the hypothetical linkage groups of the cnidarian *Nematostella vectensis* genome [2, 3]. A similar clustering of *SP* genes is found on the human chromosome D, where one member of each SP subfamily (*SP9*, *SP3* and *SP5)* is located near the *Hoxd* genes. In the known insect genomes the 3 *SP* genes have been separated; in *Drosophila melanogaster* and *Anopheles gambiae* the *SP1-4* gene is still linked to the *hox* gene cluster but the *SP6-9* and *btd* (*SP5*) genes are found on the X-chromosome [2]. The *Strigamia* genome contains three *SP* factors of which we classified one as *SP1-4*, one as *SP6-9* and one as an *SP5/buttonhead* orthologue. While we get reasonable statistical support for the grouping of SP1-4 and SP6-9 genes, the recovered SP5/btd group is poorly supported (see gene tree in add. file 2: fig. S1). We do however consider it likely that this third gene of the cluster is a true *SP5/btd* orthologue. *SP5/btd* genes are often relatively short and may lack an additional SP-box motif at the N-terminus (see gene tree in add. file 2: fig. S1 and [2])

An early assembly of the *Strigamia* genome showed chromosomal linkage of the three genes (figure S2 B, sequence of scaffold 00074/09-10 provided as suppl. file 1), a clustering that is not shown in the current publicly available assembly of the genome. Although the average scaffold size has increased from that assembly to the current, it seems more plausible that the previous one assembled this region of the genome correctly, as a coincidental mis-grouping of genes that are expected to be linked based on their evolutionary history seems rather unlikely.

Of the three factors only *SP5/btd* is expressed early in a broad domain at the anterior edge of the germ disc from where the head field develops (see above in results). *SP6-9* turns on slightly later in a segmental pattern and its expression partially overlaps with the later phase of *SP5/btd* expression (see add. file 4, fig. S3). *SP1-4* is expressed in a ubiquitous pattern throughout embryonic development (add. file 4, fig. S3), which was found similarly in other arthropods [2].

***1.2 The three centipede otx genes***

The *Strigamia* genome contains three *otx* (*otd*) paralogues, named *otx-A,* *otx-B and otx-C* [4]. *Otx-A* and *otx-B* are closely linked with only 130 bp between the 3’ end of *otx-B* and the 5’ end of *otx-A*. The pair is linked with an EH-binding protein, a microsynteny that is conserved in distantly related taxa such as vertebrates, but is lost from the *Drosophila* genome [5]. *Otx-C* does not show obvious linkage to that group, it maps onto another genomic scaffold (add. file 3, fig. S2 C). Only the otx-A predicted protein contains an additionally conserved motif (the SIWSPASI consensus motif) C-terminally of the homeodomain. In most arthropods where genomic information is available two *otd* genes were identified, of which only otd-2 predicted proteins contain this additional motif [6]. Expression of *Strigamia* *otx-A* is characterised in [7] and [8]. We collected expression data on *otx-B*, and found that both factors are co-expressed. *In situ* hybridisation using a probe against *otx-C* did not show any detectable embryonic expression.

***1.3 The centipede cap’n’collar genes***

Another case where more than one copy of a head patterning gene is present in the genome are the centipede *cnc* genes. One of the two is expressed in the characteristic mandibular ‘collar’ and anterior ‘cap’ pattern (*cnc2*, figure 3 J, K), whereas the other paralogue (*cnc1*) is expressed in a ubiquitous pattern throughout embryonic development (see add. file 5, fig. S4 C, D). *cnc1* encodes a long protein, which at its N-terminus shares sequence with the predicted protein of an ubiquitously expressed long splicing variant of the single *Drosophila* *cnc* gene [9], and with the protein of the *Tribolium* splicing variant *cnc-A*. Hence the *cnc* genes represent an example where proteins that are produced by differentially spliced transcripts of one gene in insects are encoded by gene duplicates in the myriapod [4]. We can however not exclude that the centipede *cnc* genes in addition also encode different splice variants that might be expressed at low levels.

**Table S1: list of genes and primers**

| **gene name** | **Ensembl gene ID** | **primer sequences** | **probe length** | **previously published** |
| --- | --- | --- | --- | --- |
| *ems* | SMAR007586 | right: GGAAGACAATCTTTCTTTCA  left: GCTCTATCCCGAGCATCAA | 657 bp |  |
| *otx-A* | SMAR003036 | Sequence provided by C. Brena. | 1 kb | [7, 8] |
| *otx-B* | SMAR003035 | left: AATACACTGGCGTACCACCAC right: AATCGTGACAATCAGCCAAAG | 854 bp |  |
| *otx-C* | SMAR013146 | left: ATTCCAACCGACAGGTCAAC  right:GCATAAACCGGCGATAATGA | 681 bp |  |
| *SP1-4* | SMAR004952 | right 1: TTGCTATTGGTCGTGTCAGC left: AACTCACACCCCTTTCGTTG | 1.5 kb |  |
| *SP5/btd* | SMAR004861 | left: GTGTTCGGGTGCTTTGATG right: CATTTGTTGGCGAAAATCG | 1.6 kb |  |
| *SP6-9* | SMAR004954 | left: GCATGCACACATCAACAGC right: GGTACTAGGTGCGGGTAGAGG | 1.6 kb |  |
| *col* | SMAR000841 | left: CCGTTGTGGTAAATCCGAAG right: ATCGAGTCGCGTTAAAGTCC | 1.5 kb |  |
| *cnc1* | SMAR012788 | left: AGACGTGCTTTTACGCCAAG  right: AGGTCATCGGAACTTTCAGC | 1.7 kb |  |
| *cnc2* | SMAR009751 | left: TTCAGAGTAACCAACCCATGC right: GCCAAAAGGTTCTCACGTTG | 790 bp |  |
| *hh1* | SMAR000462 | left: GTCGGTGGAAAGCATTGGCG  right: TCCCACTGCAAATAAAAATCG | 1.1 kb |  |
| *hh2* | SMAR012741 | left: AAGTTGATGCCGCTCGTGTT  right: TGTATACTGTCGCCGAACGA | 1 kb |  |
| *eng* | SMAR009094 | Sequence provided by A. Chipman. | 590 bp | [10] |
| *eve1* | SMAR012490 | Sequence provided by J. Green. | 1 kb | [11, 12] |

**References**

1. Bouwman P, Philipsen S: **Regulation of the activity of Sp1-related transcription factors.** *Mol Cell Endocrinol* 2002, **195**:27–38.

2. Schaeper ND, Prpic N-M, Wimmer EA: **A clustered set of three Sp-family genes is ancestral in the Metazoa: evidence from sequence analysis, protein domain structure, developmental expression patterns and chromosomal location.** *BMC Evol Biol* 2010, **10**:88.

3. Putnam NH, Srivastava M, Hellsten U, Dirks B, Chapman J, Salamov A, Terry A, Shapiro H, Lindquist E, Kapitonov VV, Jurka J, Genikhovich G, Grigoriev IV, Lucas SM, Steele RE, Finnerty JR, Technau U, Martindale MQ, Rokhsar DS: **Sea anemone genome reveals ancestral eumetazoan gene repertoire and genomic organization.** *Science* 2007, **317**:86–94.

4. Chipman AD, Ferrier DEK, Brena C, Qu J, Hughes DST, Schröder R, Torres-Oliva M, Znassi N, Jiang H, Almeida FC, Alonso CR, Apostolou Z, Aqrawi P, Arthur W, Barna JCJ, Blankenburg KP, Brites D, Capella-Gutiérrez S, Coyle M, Dearden PK, Du Pasquier L, Duncan EJ, Ebert D, Eibner C, Erikson G, Evans PD, Extavour CG, Francisco L, Gabaldón T, Gillis WJ, et al.: **The first myriapod genome sequence reveals conservative arthropod gene content and genome organisation in the centipede *Strigamia maritima*.** *PLoS Biol* 2014, **12**:e1002005.

5. Irimia M, Tena JJ, Alexis MS, Fernandez- Miñan A, Maeso I, Bogdanovi O, Calle-mustienes E De, Roy SW: **Extensive conservation of ancient microsynteny across metazoans due to cis -regulatory constraints Extensive conservation of ancient microsynteny across metazoans due to cis-regulatory constraints**. *Genome Res* 2012, **22**:2356–2367.

6. Browne WE, Schmid BGM, Wimmer EA, Martindale MQ: **Expression of *otd* orthologs in the amphipod crustacean, *Parhyale hawaiensis*.** *Dev Genes Evol* 2006, **216**:581–595.

7. Steinmetz P, Urbach R, Posnien N, Eriksson J, Kostyuchenko R, Brena C, Guy K, Akam M, Bucher G, Arendt D: ***Six3* demarcates the anterior-most developing brain region in bilaterian animals.** *Evodevo* 2010, **1**:14.

8. Brena C: **Myriapoda**. In *A. Wanninger (ed.), Evolutionary Developmental Biology of Invertebrates 3: Ecdysozoa I: Non-Tetraconata*; 2015:141–189.

9. McGinnis N, Ragnhildstveit E, Veraksa A, McGinnis W: **A cap “n” collar protein isoform contains a selective Hox repressor function.** *Development* 1998, **125**:4553–64.

10. Chipman AD, Arthur W, Akam M: **Early development and segment formation in the centipede, *Strigamia maritima* (Geophilomorpha).** *Evol Dev* 2004, **6**:78–89.

11. Green J, Akam M: **Evolution of the pair rule gene network: Insights from a centipede.** *Dev Biol* 2013:1–11.

12. Brena C, Akam M: **An analysis of segmentation dynamics throughout embryogenesis in the centipede *Strigamia maritima*.** *BMC Biol* 2013, **11**:112.

13. Rutherford K, Parkhill J, Crook J, Horsnell T, Rice P, Rajandream M a, Barrell B: **Artemis: sequence visualization and annotation.** *Bioinformatics* 2000, **16**:944–5.

**Additional files**

**Add. file 2, fig. S1:** Maximum likelihood gene tree of arthropod, cnidarian, mouse, zebrafish and human SP factors, unrooted. Tree based on “muscle” protein alignment of the conserved zinc finger region. An *SP6-9* clade (only to the exclusion of vertebrate and cnidarian *SP6* genes) has good bootstrap support (76/100). Within this clade *Strigamia* *SP6-9* (SMAR004954) clusters with the remaining arthropod *SP6-9* genes. All SP1-4 proteins, including *Strigamia* SP1-4 (SMAR004952) group together; this clade has 65/100 bootstrap support. *Strigamia* *SP5* (SMAR004861) forms a clade with other *SP5* and *btd* genes, only to the exclusion of *Folsomia SP5*. The SP5 group is not statistically robust though (27/100). Mm=*Mus musculus* (*Mm_SP1*: NP_038700.2; *Mm_SP2*: CAM21905.1; *Mm_SP3*; AAX90615.1; *Mm_SP4*: NP_033265.3; *Mm_SP5*: NP_071880.1; *Mm_SP6*: NP_112460.1; *Mm_SP7*: NP_569725.1; *Mm_SP8*: NP_796056.2; *Mm_SP9*: NP_001005343.1), Hs=*Homo sapiens* (*Hs_SP1*: NP_612482.2; *Hs_SP2*: NP_003101.3; *Hs_SP3*: NP_003102.1; *Hs_SP4*: NP_003103.2; *Hs_SP5*: NP_001003845.1; *Hs_SP6*: NP_954871.1; *Hs_SP7*: NP_690599.1; *Hs_SP8*: NP_874359.2; *Hs_SP9*: NP_001138722.1), Dr=*Danio rerio* (*Dr_SP1*: NP_997827.1; *Dr_SP2*: NP_001093452.1; *Dr_SP3*: NP_001082967.1; *Dr_SP4*: NP_956418.1; *Dr_SP5*: NP_851304.1; *Dr_SP6*: NP_991195.1; *Dr_SP7*: NP_998028.1; *Dr_SP8*: NP_991113.1; *Dr_SP9*: NP_998125.2), Dm=*Drosophila melanogaster* (*Dm_CG5669*: AAF56261.1; *Dm_btd*: NP_511100.1, *Dm_Sp69*: NP_572579.2), Fc=*Folsomia candida* (*Fc_SP14*: CBH30974.1; *Fc_Sp5*: FN562986; *Fc_Sp6-9*: FN562987), Tc=*Tribolium castaneum* (*Tc_SP1-4*: XP_972252.1; *Tc_btd*: NP_001107792.1; *Tc_Sp-like*: NP_001034509.1), Nv=*Nematostella vectensis* (*Nv_SP1-4*: XP_001635004.1; *Nv_SP5*: XP_001635002.1; *Nv_SP6-9*: XP_001634948.1), Gm=*Glomeris marginata* (*Gm_btdI*: CAK50835.1), Ph=*Parhyale hawaiensis* (*Ph_Sp14*: CBH30980.1; *Ph_Sp6-9*: FN562992.1).

**Add. file 3, fig. S2:** Genomic organisation and protein structure of head patterning genes. **A** Protein structure of the three centipede SP factors. The SP box motif is missing in the short SP5 protein. **B** Conserved centipede SP gene cluster. **C** Close linkage of *otx-A* and *otx-B*, and conserved microsynteny with an EH-domain-binding protein1. *otx-C* maps onto a different genomic scaffold.

**Add. file 4, fig. S3:** Expression of *SP6-9* (A-D) and *SP1-4* (E-H) during early Strigamia development. A-F, H: ventral views, G: lateral view. **A** (stage 2.2, early) *SP6-9* is expressed in the posterior hemisphere of the embryo. **B** (stage 2.2 late) *SP6-9* is expressed in anterior segmental domains where is overlaps with *SP5* expression (compare figure 2) and in a broad domain at the posterior pole. **C** (stage 2.3) and **D** (stage 3.1) Segmental expression of *SP6-9* and expression within the posterior growth zone. **E** (stage 2.2, early) *SP1-4* is uniformly expressed during blastoderm stage, a seemingly stronger expression in the posterior is due to the higher cell density in this area. **F** and **G** (stage 2.3, late; G is a lateral view of same specimen). Expression is uniform, darker areas are correlated with the germ band and with areas of multi-layered tissue/high cell density. **H** (stage 4.3) *SP1-4* expression at a mid-segmentation stage. Expression is stronger in the germ band than in the extra-embryonic territory, but largely reflects the morphology. **oc**= ocular domain, **ant/int**= antennal and intercalary expression, **md**= mandibular domain, **mx1**= 1^st^ maxillary domain.

**Add. file 5, fig. S4:** Expression of *otx-B* (single stains) and *cnc1*. All in ventral view. **A** (stage 2.2, early) *otx-B* is expressed in an anterior cap and more strongly at the posterior margin of its expression domain. **B** (stage 2.3) *otx-B* expression is strong in an ocular domain and weaker in the prospective head field anterior to that. Expression is also seen along the midline. **C** (stage 2.3, late) and **D** (stage 4.1) *cnc1* is expressed in a ubiquitous pattern, darker staining in areas of dense tissue. **oc**= ocular domain, **ml**= midline.

**Add. file 6: >scaffold00074_ass-09-10_SP-genes.fasta**< Sequence of the genomic scaffold of a previous unpublished genome assembly (from 09-10), on which the three centipede SP factors are syntenic. Mapped is the complete known cDNA sequence of *SP5*, and basepairs 1-2103 of *SP1-4*, and basepair 1-1823 of *SP6-9*. The latter two contain sequence at their 3’ ends that does not map to the scaffold (these sequences would most likely be found in stretches of the scaffold where unknown sequence is completed with an ‘N’ estimate of the gap size.) The file was created and can be read with the software Artemis [13], or similar genome viewers.
