# Supplementary figures and images for: Formation and subdivision of the head field in the centipede Strigamia maritima, as revealed by the expression of head gap gene orthologues and hedgehog dynamics
Source: EvoDevo. 2017 Oct 23;8:18. doi: 10.1186/s13227-017-0082-x (PMC5654096; doi:10.1186/s13227-017-0082-x)

Figure S1

0.1

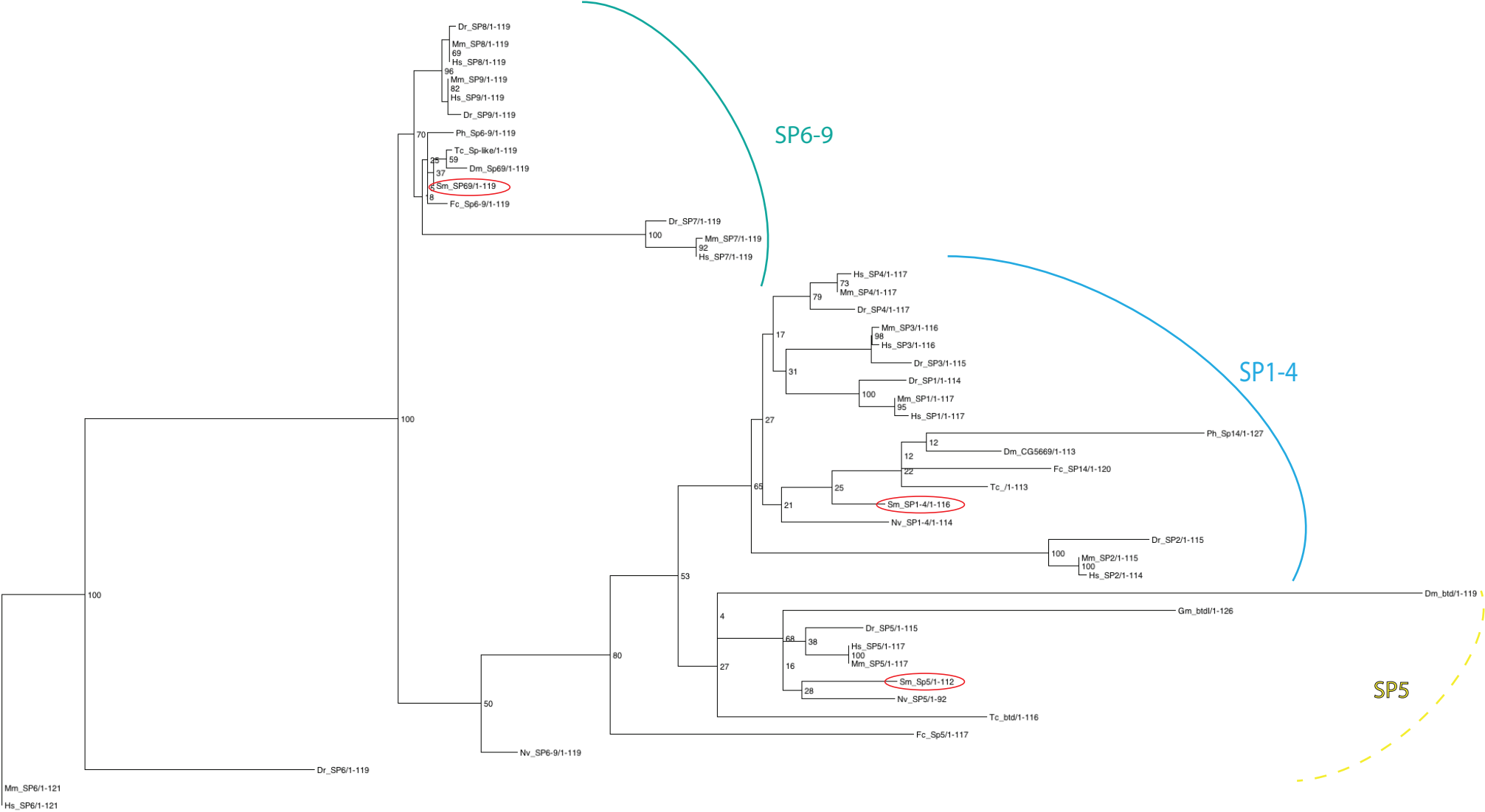

Supplement: Supplementary file 2 — Additional file 2: Fig. S1. Maximum likelihood gene tree of arthropod, cnidarian, mouse, zebrafish and human SP factors, unrooted. Tree based on “muscle” protein alignment of the conserved zinc finger region. An SP6-9 clade (only to the exclusion of vertebrate and cnidarian SP6 genes) has good bootstrap support (76/100). Within this clade Strigamia SP6-9 (SMAR004954) clusters with the remaining arthropod SP6-9 genes. All SP1-4 proteins, including Strigamia SP1-4 (SMAR004952) group together; this clade has 65/100 bootstrap support. Strigamia SP5 (SMAR004861) forms a clade with other SP5 and btd genes, only to the exclusion of Folsomia SP5. The SP5 group is not statistically robust though (27/100). Mm=Mus musculus (Mm_SP1: NP_038700.2; Mm_SP2: CAM21905.1; Mm_SP3; AAX90615.1; Mm_SP4: NP_033265.3; Mm_SP5: NP_071880.1; Mm_SP6: NP_112460.1; Mm_SP7: NP_569725.1; Mm_SP8: NP_796056.2; Mm_SP9: NP_001005343.1), Hs=Homo sapiens (Hs_SP1: NP_612482.2; Hs_SP2: NP_003101.3; Hs_SP3: NP_003102.1; Hs_SP4: NP_003103.2; Hs_SP5: NP_001003845.1; Hs_SP6: NP_954871.1; Hs_SP7: NP_690599.1; Hs_SP8: NP_874359.2; Hs_SP9: NP_001138722.1), Dr=Danio rerio (Dr_SP1: NP_997827.1; Dr_SP2: NP_001093452.1; Dr_SP3: NP_001082967.1; Dr_SP4: NP_956418.1; Dr_SP5: NP_851304.1; Dr_SP6: NP_991195.1; Dr_SP7: NP_998028.1; Dr_SP8: NP_991113.1; Dr_SP9: NP_998125.2), Dm=Drosophila melanogaster (Dm_CG5669: AAF56261.1; Dm_btd: NP_511100.1, Dm_Sp69: NP_572579.2), Fc=Folsomia candida (Fc_SP14: CBH30974.1; Fc_Sp5: FN562986; Fc_Sp6-9: FN562987), Tc=Tribolium castaneum (Tc_SP1-4: XP_972252.1; Tc_btd: NP_001107792.1; Tc_Sp-like: NP_001034509.1), Nv=Nematostella vectensis (Nv_SP1-4: XP_001635004.1; Nv_SP5: XP_001635002.1; Nv_SP6-9: XP_001634948.1), Gm=Glomeris marginata (Gm_btdI: CAK50835.1), Ph=Parhyale hawaiensis (Ph_Sp14: CBH30980.1; Ph_Sp6-9: FN562992.1). [file 13227_2017_82_MOESM2_ESM.pdf]

Figure S2

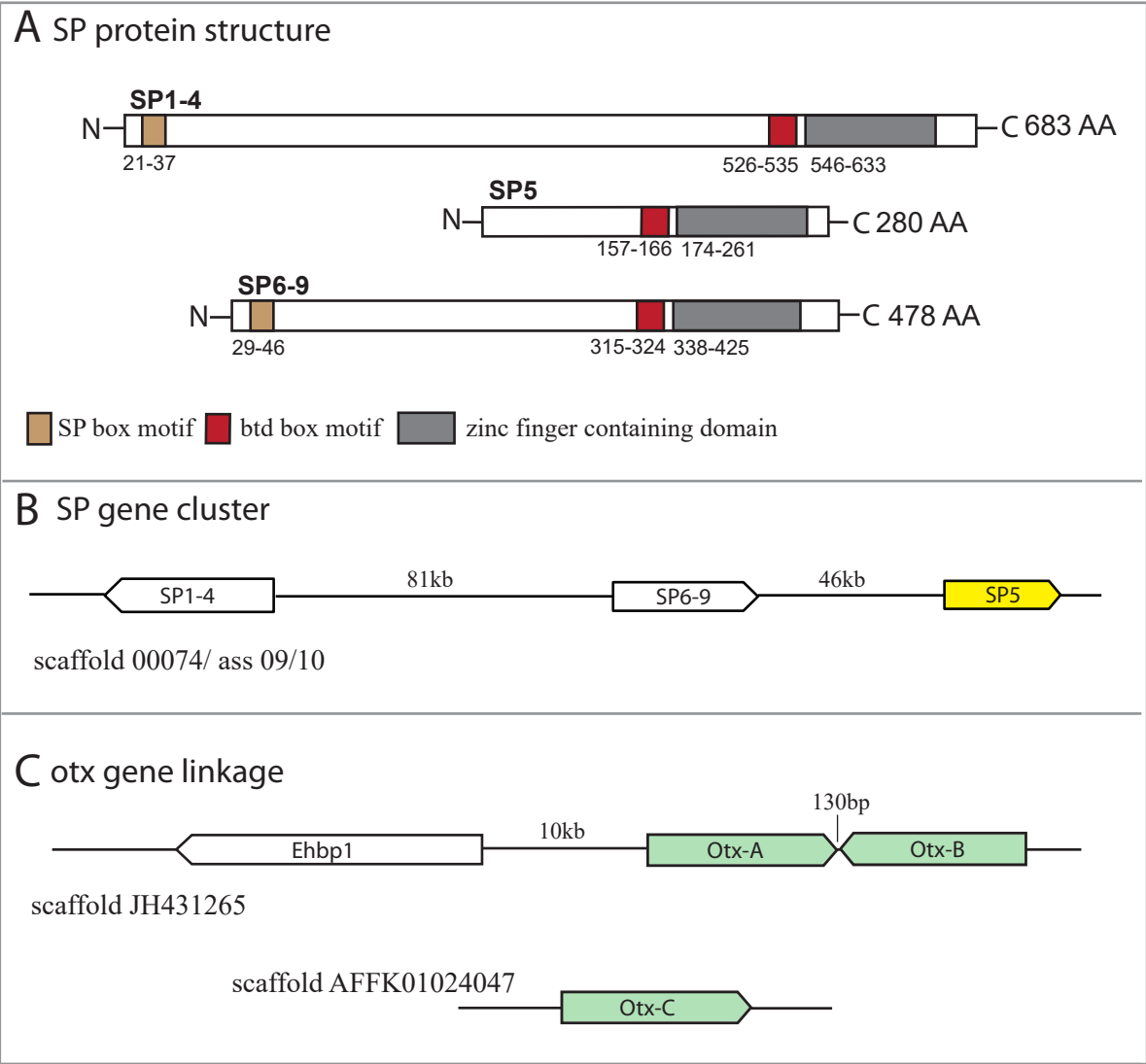

Supplement: Supplementary file 3 — Additional file 3: Fig. S2. Genomic organisation and protein structure of head patterning genes. A Protein structure of the three centipede SP factors. The SP box motif is missing in the short SP5 protein. B Conserved centipede SP gene cluster. C Close linkage of otx-A and otx-B, and conserved microsynteny with an EH-domain-binding protein 1. otx-C maps onto a different genomic scaffold. [file 13227_2017_82_MOESM3_ESM.pdf]

Figure S3

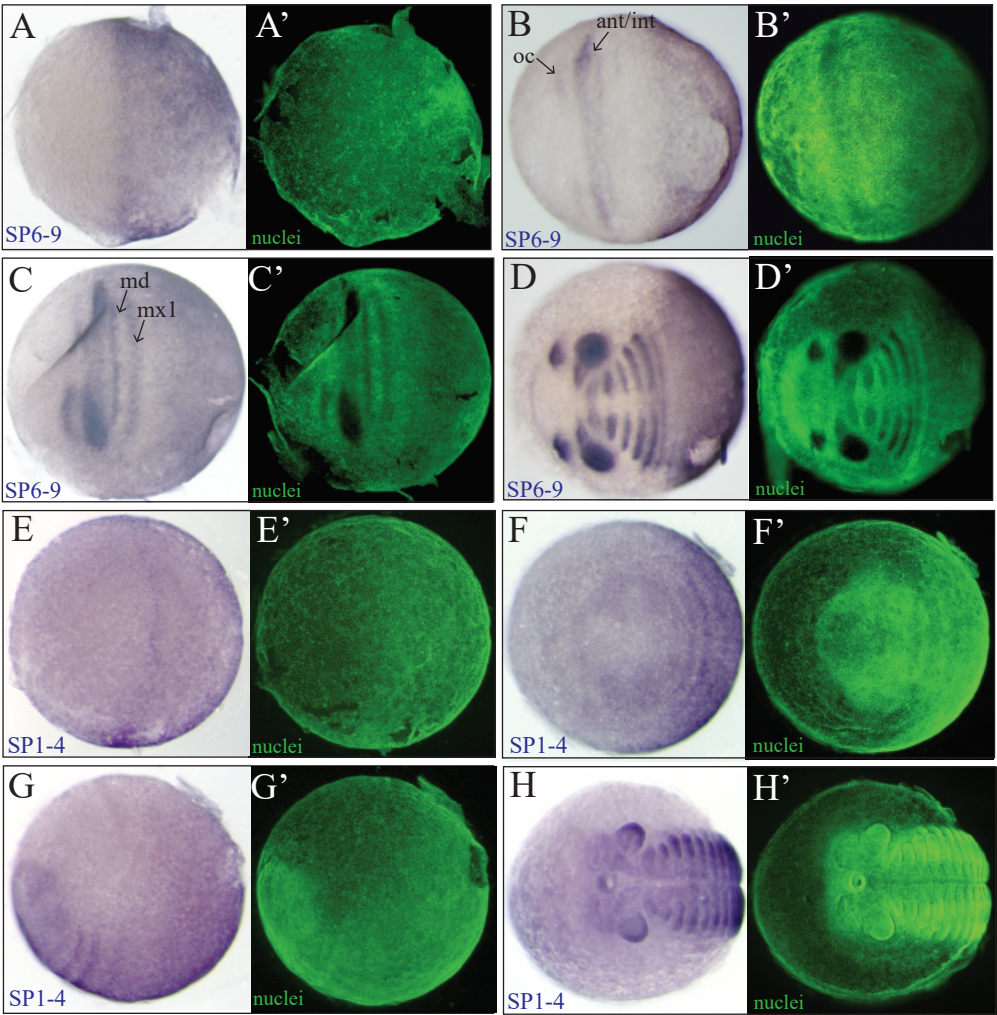

Supplement: Supplementary file 4 — Additional file 4: Fig. S3. Expression of SP6-9 (A-D) and SP1-4 (E-H) during early Strigamia development. A-F, H: ventral views, G: lateral view. A (stage 2.2, early) SP6-9 is expressed in the posterior hemisphere of the embryo. B (stage 2.2 late) SP6-9 is expressed in anterior segmental domains where is overlaps with SP5 expression (compare figure 2) and in a broad domain at the posterior pole. C (stage 2.3) and D (stage 3.1) Segmental expression of SP6-9 and expression within the posterior growth zone. E (stage 2.2, early) SP1-4 is uniformly expressed during blastoderm stage, a seemingly stronger expression in the posterior is due to the higher cell density in this area. F and G (stage 2.3, late; G is a lateral view of same specimen). Expression is uniform, darker areas are correlated with the germ band and with areas of multi-layered tissue/high cell density. H (stage 4.3) SP1-4 expression at a mid-segmentation stage. Expression is stronger in the germ band than in the extra-embryonic territory, but largely reflects the morphology. oc= ocular domain, ant/int= antennal and intercalary expression, md= mandibular domain, mx1= 1st maxillary domain. [file 13227_2017_82_MOESM4_ESM.pdf]

Figure S4

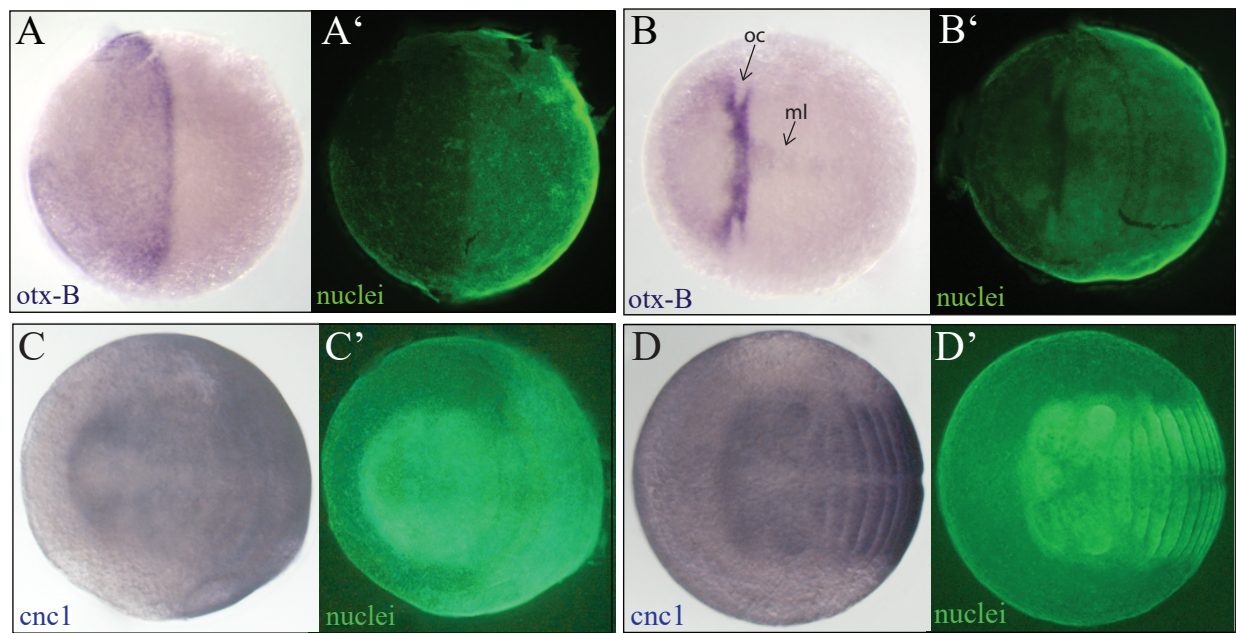

Supplement: Supplementary file 5 — Additional file 5: Fig. S4. Expression of otx-B (single stains) and cnc1. All in ventral view. A (stage 2.2, early) otx-B is expressed in an anterior cap and more strongly at the posterior margin of its expression domain. B (stage 2.3) otx-B expression is strong in an ocular domain and weaker in the prospective head field anterior to that. Expression is also seen along the midline. C (stage 2.3, late) and D (stage 4.1) cnc1 is expressed in a ubiquitous pattern, darker staining in areas of dense tissue. oc= ocular domain, ml= midline. [file 13227_2017_82_MOESM5_ESM.pdf]
